# Genomic features and the transcriptional regulation of secondary metabolite biosynthesis of endophytic fungus Xylaria sp. VDL4 isolated from Vaccinium dunalianum

**DOI:** 10.1099/mgen.0.001658

**Published:** 2026-04-28

**Authors:** Lu Li, Xiaolong Yuan, Zhonghua Chen, Yi Wang, Tingwen He, Zujiang Kang, Mingwang Luo, Chengbo Peng, Qiangxin Zhang, Linhong Lu, Jiao Yao, Jiaojun Yu, Guolei Zhu

**Affiliations:** 1 Key Laboratory of State Forestry and Grassland Administration on Highly-Efficient Utilization of Forestry Biomass Resources in Southwest China, Southwest Forestry University, Kunming, PR China; 2 Laboratory of Forest Plant Cultivation and Utilization, The Key Laboratory of Rare and Endangered Forest Plants of State Forestry Administration, Yunnan Academy of Forestry and Grassland, Kunming, PR China; 3 Hubei Key Laboratory of Economic Forest Germplasm Improvement and Resources Comprehensive Utilization, Huanggang Normal University, Huanggang, Hubei, PR China; 4 College of Forestry, Southwest Forestry University, Kunming, Yunnan, PR China

**Keywords:** biosynthesis gene cluster, transcriptional regulation, secondary metabolite, *Xylaria *sp., whole-genome sequence

## Abstract

As a prominent group of endophytic fungi, *Xylaria* species have attracted considerable research interest due to their capacity to produce diverse bioactive secondary metabolites. In this study, we performed comprehensive whole-genome sequencing and annotation of *Xylaria* sp. VDL4 using third-generation sequencing technology, followed by comparative genomic analysis. Integrated multi-omics analyses were employed to investigate the secondary metabolites and their biosynthetic pathways in *Xylaria* sp. VDL4 under various culture conditions. The results of genomic analysis showed that the genome of *Xylaria* sp. VDL4 spanned 48.5 Mb, comprised 56 scaffolds with a GC content of 51.77%. Gene prediction annotated 12,457 protein-coding genes, including 76 biosynthesis gene clusters. Among these, we identified 31 polyketide synthase (PKS) genes, 17 terpene synthase (TPS) genes and 16 non-ribosomal peptide synthase (NRPS) genes, in addition to 171 transcription factors. Phylogenomic analysis showed that *Xylaria* sp. VDL4 and *Xylaria arbuscula* B clustered in the same branch, and the divergence time was about 62.8 million years ago. Principal component analysis showed that the differential metabolites could be divided into three distinct groups: one for MSF, one for DM and a third group comprising CK and BWS. No significant difference was observed between the BWS and CK treatments, indicating that valproic acid did not exert a notable regulatory effect on secondary metabolite accumulation. Metabolomics analysis revealed that cytochalasin D and ganoderic acid Mf increased ~61.5-fold in solid cultures supplemented with tapioca compared with yeast malt broth cultures, while 10-hydroxycamptothecin was highly expressed under rice culture conditions. Transcriptomic analysis showed that five PKS genes and five TPS genes exhibited higher expression levels under solid culture conditions (DM and MSF), while three PKS and two TPS genes were more actively expressed under liquid culture conditions (CK and MY-BWS). Integrated with the multi-omics analysis, it was speculated that *Xylaria* sp. VDL4 polyketide synthase – non-ribosomal peptide synthetase 2 (*XyPKS-NRPS2*) gene – may be responsible for the biosynthesis of cytochalasin D, *XyPKS-NRPS1* may catalyse the synthesis of swainsonine and *XyPKS30* gene may catalyse the synthesis of 6-methylsalicylic acid. *Xy-TPS11* may play a key role in the biosynthesis pathway of 10-hydroxycamptothecin, and *Xy-TPS4* is related to the biosynthesis of ganoderic acid Mf. The present study provides new insights into the biosynthetic mechanism of secondary metabolites in *Xylaria* and provides an important theoretical basis for subsequent related studies.

**Impact Statement**

This study provides a comprehensive genomic characterization of the endophytic fungus *Xylaria* sp. VDL4 using whole-genome sequencing and multi-omics analysis. It focuses on deciphering the genomic features of *Xylaria* sp. VDL4, with particular emphasis on the transcriptional regulation of secondary metabolite biosynthesis, including cytochalasin D and 10-hydroxycamptothecin. The findings offer valuable insights for future molecular validation of biosynthetic gene functions and lay the foundation for exploiting this strain’s potential bioactive compounds.

## Data Summary

The Internal Transcribed Spacer (ITS) sequence of *Xylaria* sp. VDL4 has been deposited in GenBank under accession number MH305438.1, with the associated BioSample record SAMN47950275, and the complete genome assembly is available under accession number JBNCFN000000000 and SRA accession numbers SRR33321337, SRR33321336, SRR33321335 and SRR33321334 (https://www.ncbi.nlm.nih.gov/, accessed on 25 April 2025). The authors confirm that all supporting data have been provided within the article or through supplementary data files. Supplementary figures and tables can be found in the supporting information. All datasets analysed in this study are detailed in the supplementary materials.

## Introduction

Endophytic fungi are plant symbiotic fungi that inhabit healthy plants at a certain stage or throughout their life cycle and do not cause obvious disease symptoms in host plants [1]. Endophytic fungi are an important source of secondary metabolites with a variety of biological activities [2,4]. In recent years, a series of progress has been made in the study of *Xylaria* and other important families and genera [5,6].

**Fig. 1.**
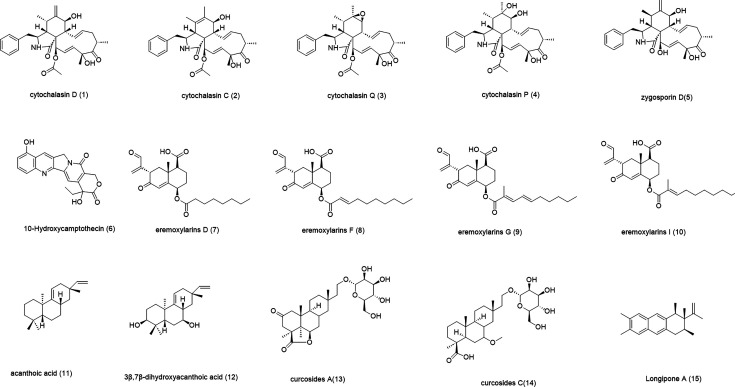
Structure diagrams of some known compounds isolated from *Xylaria*.

*Xylaria* is a genus of fungi that is widely distributed in both marine and terrestrial environments. Most species are saprophytic, growing on decaying wood and ant nests, while a few are parasitic [7]. Some fungi in the genus *Xylaria* can grow and form fruiting bodies on artificial culture media and possess certain medicinal values [8]. *Xylaria* can produce a variety of secondary metabolites with novel structures and biological activities (Fig. 1), including anti-inflammatory [9], antimicrobial [10,12], anti-tumour [13,14], antioxidant [15], cytotoxic activities [16] and *α*-glucosidase-inhibitory activities [17]. For example, cytochalasin analogues cytochalasin D (1) and cytochalasin C [2,18] with good cytotoxic activity isolated from *Xylaria* sp. NC1214, cytochalasin Q (3), with good antimelanoma activity, isolated from *Xylaria* sp. DO1801 [19], cytochalasin P (4) and zygosporin D [5,20] from *Xylaria longipes*, with cytotoxic activity and cytochalasin C [2,21], have strong acetylcholinesterase inhibitory effects. The monoterpene indole alkaloid 10-hydroxycamptothecin [6], which has anti-cancer activity, was isolated from *Xylaria* sp. YX-28 and has high efficiency, low toxicity and anticancer properties [22,24]. The anticancer activity of 10-hydroxycamptothecin is equivalent to 30 times that of camptothecin (CPT) [25]. The sesquiterpenoids eremoxylarins D (7), eremoxylarins F (8), eremoxylarins G (9) and eremoxylarins I (10) were isolated from the co-culture fermentation of *Xylaria hyperxylon* and *Dendrothyrium variisporum*, exhibited activity against three Gram-positive bacteria, including *Staphylococcus aureus*, methicillin-resistant * S. aureus* and *Staphylococcus epidermidis*. Eremoxylarins I (10) was also active against human coronavirus 229E (HCoV-229E) at a concentration nontoxic to human hepatocellular carcinoma (Huh-7) cells [26]. The compounds acanthoic acid (11) and 3*β*,7*β*-dihydroxyacanthoic acid (12) were isolated from the fungus *Xylaria* sp. (EJCP07). Among them, 3*β*,7*β*-dihydroxyacanthoic acid (12) exhibited antibacterial activity against *Bacillus subtilis* and *Escherichia coli* [27]. Additionally, the isopimarane-type diterpenoids xylarcurcoside A (13) and xylarcurcoside B (14), which possess anti-inflammatory properties, were obtained from *Xylaria curta* YSJ-5. Furthermore, the polyketide longipone A (15), displaying moderate cytotoxicity, was isolated from *Xylaria longipes* [28].

Cytochalasans are one of the main types of secondary metabolites in *Xylaria*. They are polyketide synthase-non-ribosomal peptide hybrid compounds (PKS-NRPS) widely distributed in fungi [29] and exhibit a range of important biological activities [18,21]. Notably, they interfere with the actin cytoskeleton of cells, thereby affecting cell movement, division and endocytosis, which ultimately disrupts normal cell growth [30,31]. Genomic analyses have revealed that the biosynthetic gene cluster for cytochalasans comprises four core genes – PKS-NRPS, trans-er, *α*/*β*-hydrolase and Diels–Alderase – along with various tailoring enzymes [32,34]. The production of these compounds is likely a key factor contributing to the anticancer effects observed in *Xylaria* spp., while also opening new avenues for anticancer drug research [35].

Camptothecin was initially discovered in *Camptotheca acuminata* (Nyssaceae), a deciduous tree endemic to China [36]. Similarly, 10-hydroxycamptothecin was identified in the endophytic fungus *Xylaria* sp. M20 [37]. Genomic analysis of *Alternaria burnsii* NCIM identified the biosynthetic genes responsible for camptothecin production [38]. These findings demonstrate that camptothecin can be biosynthesized not only in plants but also in fungi. 10-Hydroxycamptothecin exhibits remarkable anticancer activity. It specifically targets and inhibits DNA topoisomerase I, disrupting deoxyribonucleotide replication and ultimately inducing apoptosis, thereby effectively suppressing cancer cell proliferation. 10-Hydroxycamptothecin belongs to the class of monoterpenoid indole alkaloids (MIAs). It differs structurally from camptothecin by the addition of a hydroxyl group, which endows 10-hydroxycamptothecin with unique pharmacological properties [39]. In the synthesis of 10-hydroxycamptothecin, the C-10 hydroxylation reaction of camptothecin is the decisive key step. From the perspective of biosynthetic pathways, the biosynthesis of terpenoid compounds primarily proceeds via the mevalonate (MVA) pathway, while the synthesis of indole alkaloids mainly relies on the shikimate pathway. In the biosynthetic process of camptothecin, secologanin provides the terpenoid derivative for the formation of camptothecin, and tryptamine supplies the indole ring to form the quinoline ring of camptothecin. Strictosidine is an important precursor compound for the synthesis of CPT-type indole alkaloids. The precursor in the biosynthetic pathway of terpenoid indole alkaloids is strictosidine, which is generated from the condensation of tryptamine and secologanin, catalysed by strictosidine synthase (STR) [40].

Polyketide synthetase (PKS) is a key enzyme in the synthesis of polyketides [41,42]. According to the catalytic properties, PKS can be divided into four categories: non-reducing PKS (NR-PKS), partially reducing PKS (PR-PKS), highly reducing PKS (HR-PKS) and PKS-NRPS [43,44]. PKS-NRPS is the recombination and fusion of PKS and non-ribosomal peptide synthetases (NRPS). The PKS portion of PKS-NRPS usually contains the following structural domains: ketosynthase (KS), acyltransferase (AT), dehydratase (DH), methyltransferase, ketoreductase (KR) and acyl carrier protein (ACP). A single NRPS module is fused to the C-terminus of the PKS and usually includes adenosylation (A), condensation (C) and thioesterase (TE) [18,34].

This study employed an integrated multi-omics approach to comprehensively characterize the genomic and metabolic features of *Xylaria* sp. VDL4. The genomic architecture was elucidated through a hybrid sequencing strategy combining second-generation Illumina NovaSeq and third-generation Oxford Nanopore Technologies platforms, followed by systematic functional annotation. Building upon this genomic foundation, we conducted parallel metabolomic and transcriptomic profiling to investigate culture condition-dependent differential gene expression patterns, with particular emphasis on the biosynthetic pathways of pharmacologically significant compounds such as cytochalasin D and 10-hydroxycamptothecin. Notably, our genome-wide analysis identified and characterized ten distinct classes of transcription factors implicated in secondary metabolite biosynthesis, including key regulators, such as myeloblastosis (MYB), basic helix–loop–helix (bHLH), heat shock factor (HSF) and high mobility group (HMG) families. Through systematic bioinformatic interrogation, we further deciphered the putative regulatory networks governing the expression of terpene synthase (TPS) and PKS gene clusters. This research provides valuable insights for gene mining and transcriptional regulation studies of secondary metabolites in *Xylaria* fungi.

## Methods

### Microbial strains

The endophyte *Xylaria* sp. VDL4 was isolated from the healthy leaf tissues of *Vaccinium dunalianum* collected in Wuding County, Yunnan Province, China, in 2016. The strain was identified as *Xylaria* based on Internal Transcribed Spacer (ITS) sequencing, and the ITS sequence was deposited in GenBank under accession number MH305438.1.

### Culture of fungi

Firstly, the preserved endophytic fungus *Xylaria* sp. VDL4 was taken out of the refrigerator at −80 °C. After it was thawed naturally, it was inoculated on potato dextrose agar medium. Subsequently, the inoculated medium was placed in a dark environment in a temperature incubator at 26 °C with 60% humidity for 15 days before storage at 4 °C. For fungal cultivation, mycelial plugs (0.5 cm²) were uniformly excised from the colony margin and inoculated onto different culture media, followed by incubation at 28 °C in a constant-temperature incubator. The genomic sequencing strain was cultured on yeast malt broth medium. For metabolomic and transcriptomic sequencing, the liquid culture medium consisted of yeast malt broth (Qingdao Rishui Biotechnology, Qingdao, China) supplemented with BWS (ACMEC Biochemical, Shanghai, China) (20 µmol l^−1^ valproic acid) as the variable. Cultures were grown in 250-ml Erlenmeyer flasks at 28 °C under dark conditions with shaking at 150 r.p.m. Mycelia were harvested after 10 days of incubation. For solid-state fermentation, the basal medium contained 20 ml of MM medium (MM: 1.52 g l^−1^ KH₂PO₄ (Tianjin Zhiyuan Chemical Reagent, Tianjin, China), 0.52 g l^−1^ MgSO₄ (Tianjin Zhiyuan Chemical Reagent, Tianjin, China), 6 g l^−1^ NaNO₃ (Xilong Scientific, Shantou, China) and 0.52 g l^−1^ KCl (A100395-0500, Sangni Biotech, Shanghai, China), with the following variables: DM (20 g rice) or MSF (15 g cassava flour). Cultures were incubated at 25 °C for 20 days before mycelial collection. All media were sterilized by autoclaving at 121 °C for 20 min prior to use.

### Genome sequencing and assembly

After 10 days of liquid cultivation, samples of the ascomycete fungus *Xylaria* sp. VDL4 were collected and subjected to high-throughput sequencing at Frasergen Bioinformatics Co., Ltd. (Wuhan, China). The whole-genome shotgun approach was employed, utilizing 400 bp insert fragments to construct the gene library of *Xylaria* sp. VDL4. Sequencing was conducted using NGS technology on the Illumina NovaSeq platform, along with TGS technology. Raw data were processed using FastQC. The 3′-terminal DNA junctions were decontaminated using AdapterRemoval (version 2) [45], and quality correction of all reads was performed using Soapec (V2.0) software. The KMER frequency for correction was set to 17 to obtain high-quality adaptor-free genome sequences. The data were assembled *de novo* to construct contigs and scaffolds. The obtained contigs and scaffolds were corrected using Pilon v1.18 [46] software. The effect of genome assembly was evaluated by BUSCO (Benchmarking Universal Single-Copy Orthologs, http://busco.ezlab.org, v3.0.2).

### Gene prediction and annotation

Genome annotation mainly includes three research directions: repetitive sequence identification, gene structure prediction, functional annotation and non-coding RNA prediction.

#### Repeat annotation

The repetitive sequences, including tandem repeats and TEs, were searched. First, we used Tandem Repeats Finder (v4.09.1) [47] to annotate the tandem repeats using the following parameters: 2 7 7 80 10 50 2000. Then, TEs were identified at both the DNA and protein levels using a combination of *de novo* and homology-based approaches. At the DNA level, LTR_FINDER (v1.0.7) [48] was first used to identify LTR-RTs, and RepeatModeler (v2.0.1) [49] was utilized to construct a *de novo* repeat library, which comprised a repeat consensus database with classification information. We employed RepeatMasker (v4.1.2) [50] to search for similar TEs in the known Repbase TE library [51,52] and *de novo* repeat library. At the protein level, Repeat Protein Mask within the RepeatMasker package was used to search against the TE protein database using a wu-blastx engine.

#### Gene annotation

We used homologous, *ab initio* and transcriptome-assisted annotation to predict the structure of coding genes. For homologous annotation, tblastn (v2.11.0+) [53] was used to compare the related species to the reference genome. Then, the aligned sequences and their corresponding proteins were filtered and transmitted to the Exonerate (v2.4.0) [54] for accurate alignment. Augustus (v3.4.0) [55,57] and GlimmerHMM (v3.0.4) [58] were used for *de novo* annotation. For RNA-seq data, we use both *de novo* and genome-based transcriptome assemblies. RNA-seq alignments were produced using HiSat2 (v.2.2.1) [59], and then RNA-seq alignments were further assembled into transcripts with genome-guided assembler Stringtie (v.2.1.7) [60]. Additionally, the transcriptome was assembled *de novo* using Trinity (v2.8.5) [61]. We built a comprehensive transcriptome database using all transcripts from RNA-seq and Iso-seq according to the PASA pipeline (v2.4.1) [62]. Maker (v3.01.03) [63] was used to integrate the predicted gene sets into a non-redundant, more complete and reliable gene set. Finally, the PASA pipeline (v2.4.1) was used to update maker consensus predictions, adding UTR annotations and models for alternatively spliced isoforms.

#### Functional annotations

Gene functions were inferred according to the best match of the alignments to the National Center for Biotechnology Information (NCBI) Non-Redundant (NR), Kyoto Encyclopedia of Genes and Genomes (KEGG) database [64], Gene Ontology (GO) [65], TrEMBL and Swiss-Prot [66] protein databases using diamond blastp (v2.0.7) [67] with an *E*-value threshold of 1*E*−5. The protein domains were annotated using InterProScan (v5.50–84.0) [68] based on InterPro [69] protein databases.

Based on the above prediction results, the gene sets obtained through various methods of MAKER [70] were integrated into a non-redundant and more comprehensive gene set. Subsequently, PASA [62] was employed to refine the gene structures using transcriptomic data.

### Comparative genomic analysis

The whole-genome data for *Xylaria* sp. FL1042, *Xylaria arbuscula*, *Xylaria bambusicola*, *X. curta*, *Xylaria flabelliformis*, *Xylaria grammica*, *Xylaria* FL0064, *X. longipes*, *Xylaria* cf. *heliscus*, *Xylaria nigripes*, *Xylaria scruposa*, *Xylaria cubensis*, *Xylaria castorea*, *Xylaria venustula*, *Xylaria telfairii* and *Xylaria hypoxylon* were obtained from NCBI (https://blast.ncbi.nlm.nih.gov/, accessed on 17 January 2025). The antiSMASH online tool (https://antismash.secondarymetabolites.org/, accessed on 17 January 2025) was used to predict gene clusters in the scaffolds of *Xylaria* sp. VDL4. Comparative genomic and gene family analyses were subsequently performed. Protein sequences from closely related species were aligned to conduct gene family clustering, phylogenetic tree reconstruction and divergence time estimation. Species-specific genes were identified to infer evolutionary relationships, reconstruct the genomic evolutionary history of both the target species and its broader clade and estimate speciation divergence times.

#### Gene family clustering and phylogenetic analysis

Gene families were identified by clustering protein-coding genes from *Xylaria* sp. VDL4 and 16 closely related species using OrthoFinder (v2.5.4) [71]. This analysis grouped orthologous and paralogous sequences to define conserved and lineage-specific gene families. Single-copy orthologs were used to construct a phylogenetic tree. Protein sequences were aligned with muscle (v3.8.31) [72], and alignments of the corresponding coding sequences were generated and concatenated with the guidance of protein alignment. The maximum-likelihood method was used to construct the phylogenetic tree with RAxML (v8.2.12) [73].

#### Divergence time estimation

The reconstructed phylogenetic tree was used for divergence time estimation, with calibration points obtained from the TimeTree database [74] and published literature. The analysis was performed using the r8s software [75] and the mcmctree program from the PAML package [76].

### Metabolomic analysis

The metabolomics assay is divided into four groups; each group has three replicates. The quality control (QC) sample is a sample of 12 samples mixed with the same amount of sample chromatography (LC-30A, Shimadzu, Japan) and mass spectrometry (TripleTOF 6600+, SCIEX, Foster City, CA, USA). The whole process of material separation by chromatography to material identification by mass spectrometry is realized. Liquid chromatography-MS/MS (LC-MS/MS) enables accurate metabolite identification and quantification. The primary objective of metabolomic analysis is to detect and screen biologically significant metabolites exhibiting statistically significant differences from biological samples, thereby elucidating metabolic processes and underlying regulatory mechanisms in organisms.

To eliminate the potential interference of the culture medium on metabolite detection results, this study systematically excluded exogenous compounds derived from non-fungal organisms (including plants and bacteria) and artificially synthesized substances through literature database comparisons. Furthermore, fungal characteristic secondary metabolites (such as polyketides, terpenoids and alkaloids) were analysed, retaining only metabolic signals clearly consistent with fungal biosynthetic pathways. More critically, this study integrated transcriptomic and metabolomic data for cross-validation – by correlating the expression levels of key genes involved in fungal secondary metabolic pathways with the accumulation patterns of corresponding metabolites, it mutually verified the findings at the gene-metabolite level, effectively distinguishing endogenously synthesized fungal products from exogenous culture medium components. Although the aforementioned strategies have reduced the influence of culture medium interference to some extent, to completely eliminate this potential bias, blank culture medium control experiments will be supplemented in subsequent validation of biosynthetic gene processes to further verify the reliability of the conclusions drawn in this study.

#### Metabolomics data processing and statistical analysis

In this study, an untargeted metabolomics workflow was employed, encompassing data acquisition, preprocessing, metabolite annotation and statistical analysis to identify differential metabolites between experimental conditions.

#### Data preprocessing and metabolite annotation

Raw mass spectrometry data acquired in both positive (ESI+) and negative (ESI-) ion modes on an AB TripleTOF 6600+ system were converted to mzXML format using ProteoWizard. Subsequent preprocessing, including peak picking, alignment and retention time correction, was performed using XCMS. To ensure data quality, features with a missing rate >50% across samples were filtered. Missing values were imputed using the K-nearest neighbours (KNN) algorithm, and peak areas were corrected via support vector regression. Metabolite identification was conducted by querying a consolidated database encompassing a laboratory-built repository, public databases (e.g. HMDB, KEGG, Metlin), predictive databases and the metDNA method. Identifications were filtered based on a comprehensive identification score >0.5 and a coefficient of variation <0.5 in QC samples. After merging results from both ion modes, a total of 4,389 metabolites were annotated for subsequent analysis. Annotation confidence levels were defined as level 2 (putatively annotated compounds, based on MS/MS spectral matching) and level 3 (tentatively characterized compound classes, based on accurate mass matching), with all discussed differential metabolites meeting at least level 2 criteria.

#### Multivariate statistical analysis and differential metabolite screening

To explore overall metabolic profiles, unsupervised principal component analysis (PCA) was performed. Prior to PCA, the peak intensity data were normalized using unit variance scaling. PCA was implemented via the function in R (v4.1.2) to assess inter-group separation and intra-group variability.

For the targeted discovery of differential metabolites, supervised orthogonal partial least squares-discriminant analysis (OPLS-DA) was employed using the package (v1.0.1) in R. The input data were log₂-transformed and mean-centred before model construction. The robustness of the OPLS-DA model was validated through a 200-cycle permutation test. Differential metabolites were screened by combining multivariate and univariate criteria: a variable importance in projection (VIP) score >1 from the OPLS-DA model and an absolute fold change (|FC|) ≥ 2 (i.e. |log₂FC|≥1). The statistical significance of individual metabolites was further assessed using Student’s t-test, with *P*-values adjusted for multiple comparisons using the false discovery rate method. Metabolites satisfying both VIP >1 and |log₂FC|≥1 were defined as statistically significant differential metabolites.

#### UHPLC conditions

Based on the procedures described by Qiao *et al*. [77], all analyses were performed by an LC-30A system (LC-30A, Shimadzu, Japan) connected to a mass spectrometer (TripleTOF 6600+, SCIEX, Foster City, CA, USA). Chromatographic separation was performed using a Waters ACQUITY Premier HSS T3 Column (1.8 µm, 2.1 mm, 100 mm, Waters, Milford, MA, USA). The mobile phase comprised (A) H2O (containing 0.1% formic acid) (Aladdin, Shanghai, China) and (B) acetonitrile (Merck, Darmstadt, Germany) (containing 0.1% formic acid). The flow rate was 0.4 ml min^−1^. The mobile phase gradient was 0 min, 5% B; 2 min, 20% B; 5 min, 60% B; 6–7.5 min, 99% B and 7.6–10 min, 5% B. The sample preparation procedure was as follows: precisely weigh 50 mg of the sample using an electronic balance (MS105DΜ, Mettler-Toledo, Zurich, Switzerland), and add 600 µl of a 70% methanol (Merck, Darmstadt, Germany) extraction solution containing an internal standard. (The internal standard solution was prepared by dissolving 1 mg of the standard in 1 ml of a 70% methanol–water solution to obtain a 1,000 µg ml^−1^ standard stock solution, which was then further diluted with 70% methanol to prepare a 250 µg ml^−1^ internal standard working solution.) The injection volume of the sample was 4 µl. Another aliquot of the sample was analysed under negative ion mode, using the same elution gradient as in the positive ion mode.

#### MS conditions (AB)

The data acquisition was operated using the information-dependent acquisition mode using Analyst TF 1.7.1 software (Sciex, Concord, ON, Canada). The source parameters were set as follows: ion source gas 1 (GAS1), 50 psi; ion source gas 2 (GAS2), 50 psi; curtain gas (CUR), 25 psi; temperature (TEM), 550 °C; declustering potential, 60 V or 60 V in positive or negative modes, respectively; and ion spray voltage floating, 5,000 V or 4,000 V in positive or negative modes, respectively. The TOF MS scan parameters were set as follows: mass range, 50–1,000 Da; accumulation time, 200 ms; and dynamic background subtraction, on. The product ion scan parameters were set as follows: mass range, 25–1,000 Da; accumulation time, 40 ms; collision energy, 30 or 30 V in positive or negative modes, respectively; collision energy spread, 15; resolution, UNIT; charge state, 1 to 1; intensity, 100 cps; exclude isotopes within 4 Da; mass tolerance, 50 ppm; maximum number of candidate ions to monitor per cycle, 18.

### Transcriptome sequencing and differential gene expression analysis

Using NGS technology based on the Illumina HiSeq platform, we sequenced the samples under four different culture conditions using a pair-end sequencing method: liquid culture MY-CK (control), MY-BWS (supplemented with 20 µl valproic acid), solid culture DM (20 g rice in 20 ml MM medium) and MSF (15 g cassava flour in 20 ml MM medium). By referring to the sequences of PKS, TPS and transcription factors (TFs) in the genomic data, we quantified their gene expression levels in the transcriptomic data as per kilobase transcribed fragments per million mapped reads (FPKM) values. TBtools version 2.056 software was used to create interactive heatmaps to analyse the expression levels of the target genes.

### Prediction of PKS and TPS proteins

Secondary metabolite biosynthetic gene clusters (BGCs) in the *Xylaria* sp. VDL4 genome were predicted and annotated using the antiSMASH platform (v7.0), which identifies and characterizes bioactive molecule-producing gene clusters, including type I, II and III PKSs and NRPSs. antiSMASH classifies PKSs into distinct subtypes based on their architecture and functional domains. Candidate PKS gene sequences were subsequently analysed via Protein blast (https://blast.ncbi.nlm.nih.gov/, accessed on 17 January 2025) against the NCBI non-redundant database, followed by conserved domain verification. Based on domain organization, PKSs were categorized into (I) NR-PKSs, (II) PR-PKSs, (III) HR-PKSs and PKS-NRPS.

InterProScan v5.44–79.0 [68] was used to identify TPS proteins in *Xylaria* sp. VDL4 based on the associated terms for their conserved domains: terpene cyclase (IPR034686), squalene cyclase (IPR018333), polyprenyl synthetase (IPR000092), squalene synthase (IPR002060) and trichodiene synthase (IPR024652). The candidate gene sequences obtained were compared against the NCBI protein database for confirmation. Subsequently, multiple sequence alignment was conducted using DNAMAN software version 6 to identify conserved domains.

### Cluster analysis

Known PKS and TPS protein sequences were downloaded from NCBI and compared with the protein sequences from this study using the Clustal W program in the mega 5.0 software of IQ-TREE. The IQ-TREE web server quickly and accurately generates phylogenetic trees using the maximum-likelihood method (http://iqtree.cibiv.univie.ac.at/, accessed on 6 January 2025). The analysis involved 1,000 bootstrap iterations using default parameters to construct the cluster tree.

### Secondary metabolite biosynthesis gene cluster analysis

The antiSMASH platform (https://antismash.secondarymetabolites.org/, accessed on 17 January 2025) was employed to predict BGCs within the *Xylaria* sp. VDL4 genome scaffold and other fungal strains. Gene structure prediction was performed using the FGENESH online tool (https://softberry.com/, accessed on 17 January 2025). For contigs containing PKS genes, the *PKS/NRPS* Analysis Tool (https://nrps.igs.umaryland.edu/, accessed on 17 January 2025) was utilized to identify putative gene clusters and annotate functional domains. Additionally, protein sequence homology analysis was conducted using blastp (https://blast.ncbi.nlm.nih.gov/, accessed on 17 January 2025) to compare these contigs against reference databases.

### Identification and analysis of transcription factors

Domain files for MYB (PF00249), bHLH (PF00010), basic Leucine Zipper (PF00170), Homeobox (PF00046), Fungal_trans domain (PF04082), WHTH (PF00250), Zinc Finger (PF16297), Fungal Transcription Factor Domain (TFIIB) (PF08613), HSF (PF00447) and HMG (PF00505) TFs were downloaded from the InterProScan database. The protein sequences of *Xylaria* sp. VDL4 were subjected to genome-wide screening using HMMER (v3.3.2) with an *E*-value cutoff of <10^−5^. Putative transcription factors with amino acid sequences shorter than 100 residues were manually excluded from further analysis. Candidate genes were subsequently validated for conserved domains using the NCBI Conserved Domain Database (https://www.ncbi.nlm.nih.gov/Structure/cdd/wrpsb.cgi, accessed on 17 January 2025). Conserved motifs within the identified transcription factors were characterized using the MEME Suite (http://meme-suite.org/, accessed on 17 January 2025), with subsequent visualization of motif architectures and gene structures performed using TBtools (v1.09876).

### Prediction of transcription factor binding sites

Using whole-genome and transcriptome data from *Xylaria* sp. VDL4, we extracted the 2,000-bp DNA sequence upstream of the translation start codon (ATG) of *Xylaria* sp. VDL4 polyketide synthase (*XyPKS*) and *Xylaria* sp. VDL4 terpene synthase (*XyTPS*) genes that exhibited expression patterns similar to those of candidate TFs. This sequence extraction was performed using TBtools (v1.108). Subsequently, potential transcription factor binding sites within the promoter regions of these co-expressed genes were predicted using the JASPAR database (2024 release), with a confidence threshold set at 90% [78].

### Real-time quantitative fluorescence PCR

Genes exhibiting similar expression patterns under various cultivation conditions were selected, including *XyTFs*, *XyPKS* and *XyTPS* genes, such as *XyPKS2*, *XyPKS30*, *XyTPS7*, *XyTPS8*, *XyTPS12*, *XyAnk_2–34*, *XyAnk2-68*, *XyBerberine*, *XybHLH3* and *XyMYB5*. Primers were designed using Primer Premier 5.0 software to detect the expression of target genes (Table S5). Quantitative real-time PCR was performed to measure gene expression levels. Each 20 µl reaction mixture contained 10 µl of PCR master mix, 1 µl of DNA/cDNA template, 2 µl of primers and 7 µl of nuclease-free water. The thermal cycling protocol consisted of an initial denaturation step (94 °C for 2 min), followed by 40 amplification cycles (94 °C for 15 s, 65 °C for 15 s and 72 °C for 45 s), with a final extension at 72 °C for 10 min. All quantitative real-time PCR assays were performed in triplicate, and statistical analysis was conducted using SPSS software.

## Results

### Basic features of the *Xylaria* sp. genome

#### Genome annotation

Illumina sequencing produced 31,073,882 high-quality reads, yielding a draft genome assembly of *Xylaria* sp. VDL4 with a total size of 48.5 Mb. The assembly comprised 56 scaffolds, with the longest scaffold measuring 6,068,945 bp and an N50 scaffold length of 5,185,669 bp. The overall GC content was 51.77%. A total of 12,457 protein-coding genes were predicted, with an average coding sequence length of 16,061 bp (Table 1). For non-coding RNA annotation, tRNA genes were identified using tRNAscan-SE (v2.0) [79] based on their characteristic cloverleaf secondary structures. rRNA genes were predicted using RNAmmer v1.2 with default parameters. In addition, miRNA and snRNA sequence information on the genome can be predicted using the covariance model of the Rfam (http://pfam.xfam.org/, accessed on 17 January 2025) [80] family using Rfam’s own INFERNAL [81] software. This analysis used tRNAscan-SE, RNAmmer and Rfam_scan to predict the secondary structure of 198 tRNAs, 74 rRNAs and 18 snRNAs, respectively.

**Table 1.** Genome assembly of *Xylaria* sp. VDL4

Item	Value	Item	Value
Total sequence length	50,958,917	Scaffolds	56
Total length (Mb)	48.5	Contigs	56
Max length (bp)	6,068,945	Scaffolds N20	5,549,173
GC content (%)	51.77	Contigs N20	5,549,173
Gene number	12,457	Scaffolds N50	5,185,669
Total gene length	20,007,360	Contigs N50	5,185,669
Gene/genome (%)	39.26	Scaffolds N90	3,938,258
Average gene length (bp)	1606.1	Contigs N90	3,938,258

Basic characteristics of the genome of Xylaria sp. VDL4

bp, base pair; GC, guanine-cytosine; Mb, million base pair.

#### The gene functions of *Xylaria* sp. VDL4

The 12,457 non-redundant genes predicted in the *Xylaria* sp. VDL4 genome were functionally annotated using multiple databases: InterPro, NR, GO, KEGG, EggNOG, Swiss-Prot and TrEMBL. Annotation results showed that 7,026, 12,053, 7,516, 10,449, 3,963, 8,042 and 9,549 genes were assigned functions in these databases, respectively. The NR database provided the highest annotation coverage, with 96.76% of all predicted genes being successfully annotated (Table 2).

**Table 2.** Functional annotation of the *Xylaria* sp. VDL4 genome

Database	No.	Percentage (%)
InterPro	7,026	69.42
NR	12,053	96.76
GO	7,516	60.33
EggNOG	10,449	83.88
KEGG	3,963	31.81
Swissprot	8,042	64.56
TrEMBL	9,549	94.35

**Fig. 2.**
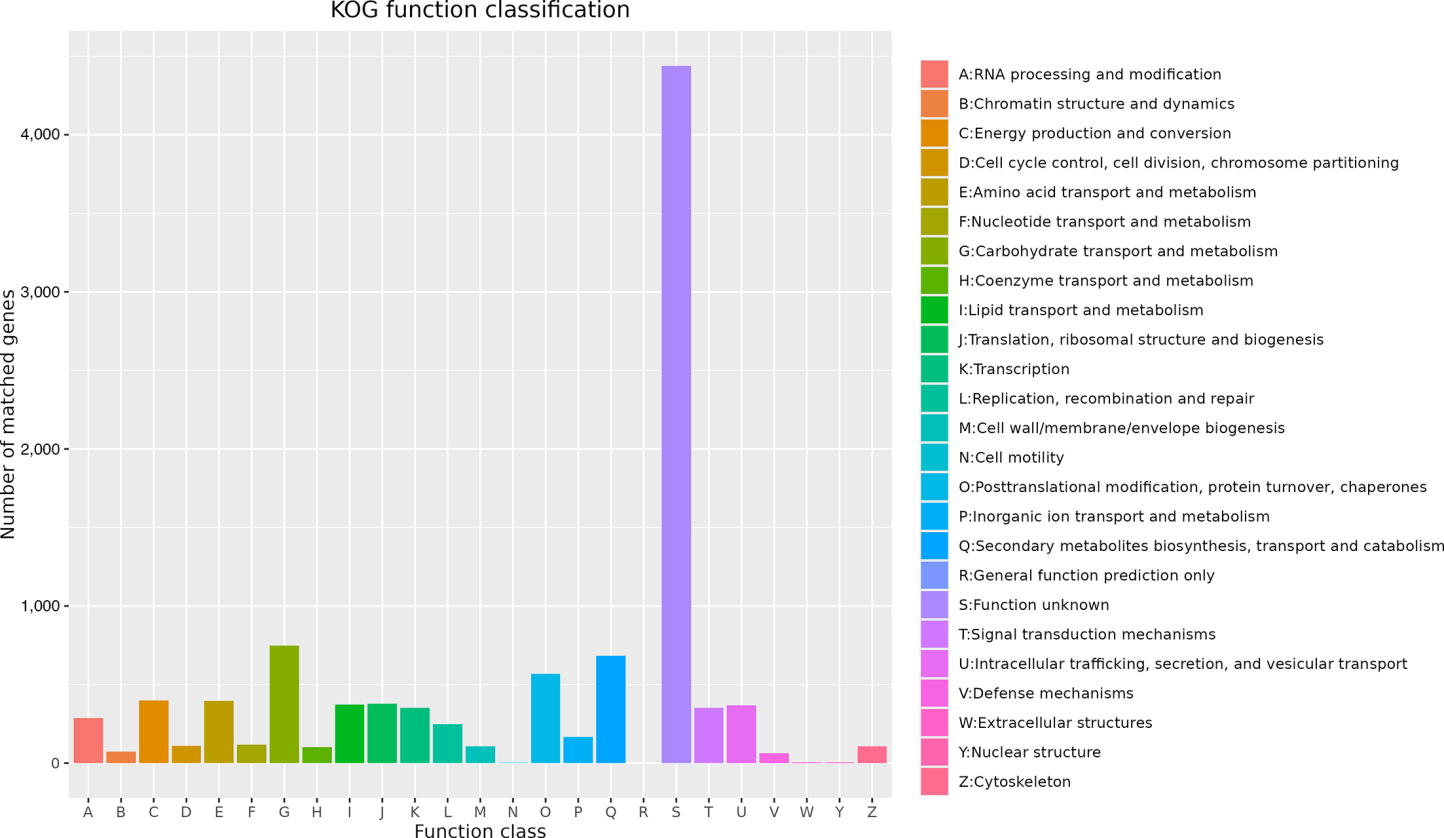
Functional annotation of EggNOG analysis of *Xylaria* sp. VDL4 genes encoding the proteins. The *x*-axis represents the annotated gene categories, while the *y*-axis indicates the gene counts. The horizontal axis represents the number of annotated genes under each pathway category; the horizontal axis represents the number of annotated genes under each pathway category; the vertical axis represents the pathway classifications. Different colours indicate the broader categories to which they belong.

EggNOG annotation showed that the most abundant gene categories were ‘Function unknown’ (4,439), ‘Carbohydrate transport and metabolism’ (749), ‘Secondary metabolites biosynthesis, transport and catabolism’ (682) and ‘Posttranslational modification, protein turnover, chaperones’ (568) (Fig. 2).

The annotations in the GO database allow us to gain insight into the biological significance represented by the genes. GO functional analysis shows (Fig. S1, available in the online Supplementary Material) that the annotated genes are distributed across three functional categories: biological process, cellular component and molecular function. The categories include ‘biological process’ (6,888); ‘cellular nitrogen compound metabolic process’ (1,761) and ‘biosynthetic process’ (1,602) from biological processes; ‘cell’ (2,638), ‘intracellular’ (2,537) and ‘cellular-component’ (2,183) from cellular components and ‘molecular-function’ (6,438), ‘ion binding’ (2,677) and ‘oxidoreductase activity’ (1,448) from molecular function.

KEGG enrichment analysis showed that the genes were mainly enriched in pathways for genetic information processing (2,427), signalling and cellular processes (664), signal transduction (564), metabolism (550), carbohydrate metabolism (452) and amino acid metabolism (413) (Fig. S2). The gene abundance data reflect the relative activity levels and biological significance of various metabolic pathways within the cell.

#### Additional annotation of *Xylaria* sp. VDL4

**Fig. 3.**
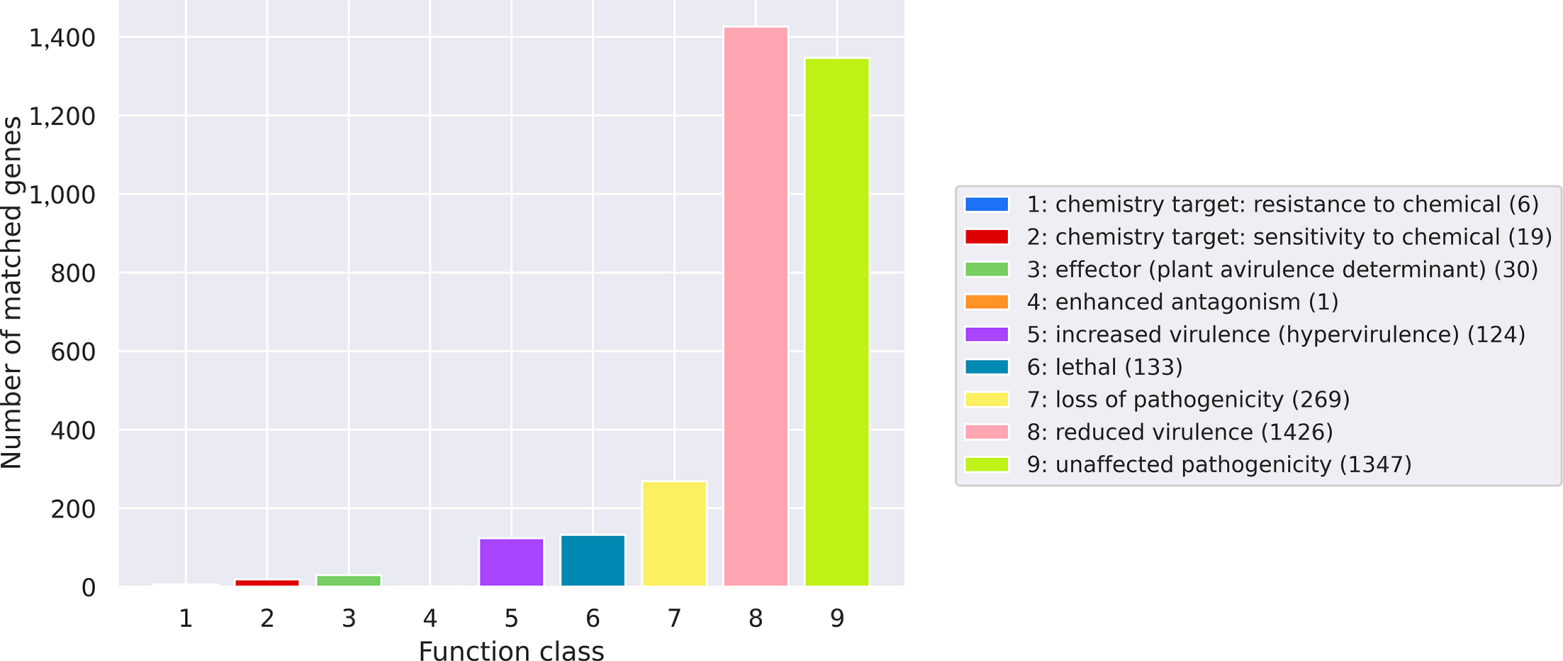
Distribution map of mutation types in the pathogen PHI phenotype of *Xylaria* sp. VDL4. The *x*-axis represents the annotated gene categories, while the *y*-axis indicates the gene counts. The horizontal axis represents the number of annotated genes under each pathway category; the horizontal axis represents the number of annotated genes under each pathway category; the vertical axis represents the pathway classifications. Different colours indicate the broader categories to which they belong.

The Pathogen–Host Interactions database (PHI) categorizes pathogen phenotypic mutations, with the gene count distribution across mutation types shown in Fig. 3. *Xylaria* sp. VDL4 harbours a diverse set of PHI-base genes, including reduced virulence (1,426), unaffected pathogenicity (1,347), loss of pathogenicity (269), lethal (133), increased virulence (hypervirulence) (124), effector (plant avirulence determinant) (30), resistance to chemicals (6) and sensitivity to chemicals (19).

#### Transporter classification database

**Fig. 4.**
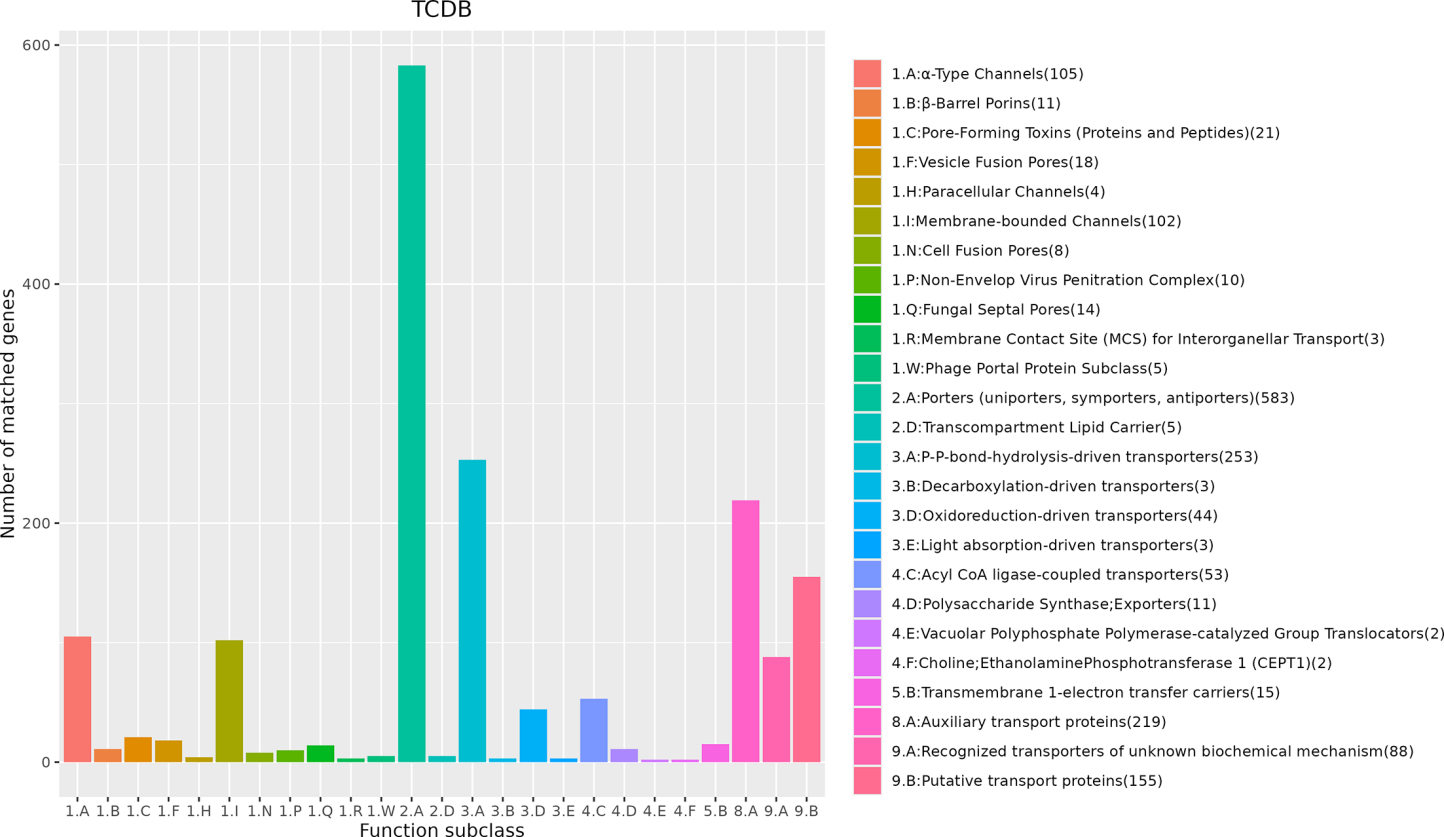
CAZy functional classification chart of *Xylaria* sp. VDL4. The *x*-axis represents the annotated gene categories, while the *y*-axis indicates the gene counts. The horizontal axis represents the number of annotated genes under each pathway category; the horizontal axis represents the number of annotated genes under each pathway category; the vertical axis represents the pathway classifications. Different colours indicate the broader categories to which they belong.

The Transporter Classification Database is a freely accessible reference for transport protein research, offering structural, functional, mechanistic, evolutionary and disease-related information on transporters from various organisms. Analysis shows that the strain contains a diverse array of cell membrane transport proteins, including 247 primary active transporters, 303 electrochemical potential-driven transporters, 588 channels/pores, 301 accessory factors involved in transport, 219 incompletely characterized transport systems, 243 group translocators and 68 and 15 transmembrane electron carriers (Fig. 4). This suggests a broad capacity and functional diversity for substance transport in this strain.

### Genomic characteristics of 16 strains

The genome of *Xylaria* sp. VDL4 was characterized and compared with 16 related species (Table S1). The genome of *X. grammica* was the largest (54.7 Mb), while that of *X. nigripes* was the smallest (24.6 Mb). Due to the differences in sequencing platforms and assembly techniques, the number of scaffolds varied among the genomes. *Xylaria* sp. FL1042 had the most scaffolds (2,489), while *X. grammica* had the fewest (25). The GC content of the genomes of the 16 strains ranged from 39.5 to 48.5%.

### Comparative genomic analysis

#### Clustering of gene families

To determine the gene families of protein-coding genes, OrthoFinder (v2.5.4) [71] was used to cluster proteins. By constructing gene families, single-copy gene families and multi-copy gene families are obtained, which are relatively conserved among species. Single-copy gene families have only one gene copy in all species, while multi-copy gene families have experienced multiple duplications during evolution, particularly in higher organisms. Some of these repetitive DNA sequences continue to undergo evolutionary divergence and become new genes different from the original sequence. Some of them remain in the same form of structure and function and become multi-copy gene families (Fig. S3). The clustering results of the first four species (Table S1) were taken, and the Venn diagram was drawn. From the Venn diagram, it can be seen that the number of gene families shared by *Xylaria* sp. VDL4 with the other three species is 5,426, and the number of unique gene families of *Xylaria* sp. VDL4 single species relative to the other three species is 664 (Fig. S4).

### Phylogenetic analysis

**Fig. 5.**
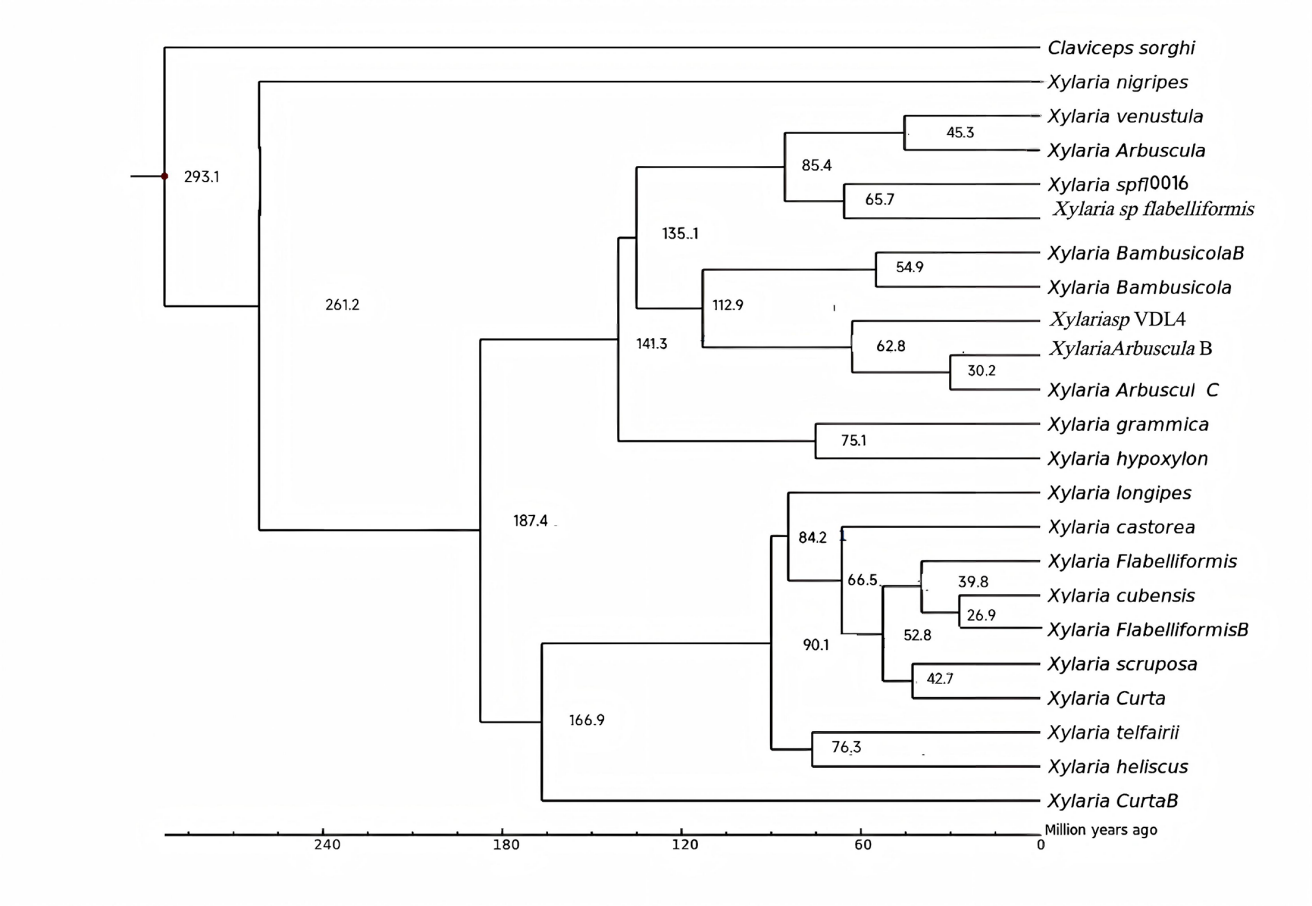
Divergence time phylogenetic tree of *Xylaria* sp. VDL4, the peripheral species *Claviceps sorghi* and 16 related species. Note: Each branch represents a specific *Xylaria* species or species group. The numerical values on the branches (e.g. 100) indicate the branch support values (typically bootstrap percentages). The horizontal axis represents genetic divergence (in substitutions per site), with larger values indicating greater genetic differences between species.

The phylogenetic tree constructed through gene family clustering elucidates the evolutionary relationships among different *Xylaria* species. Single-copy orthologs were used to construct a phylogenetic tree. There are 1,874 single copies of orthologous genes. Protein sequences were aligned with muscle (v3.8.31) [73], and alignments of the corresponding coding sequences were generated and concatenated with the guidance of protein alignment. Phylogenetic trees of these genes were constructed using RAxML [82] (Fig. S5). The phylogenetic analysis revealed that these species were divided into several major clades, each representing a specific *Xylaria* species or species group. The numerical values on the branches indicate the support values for those clades, typically calculated using the bootstrap method. Values approaching 100% suggest high confidence in the clustering results. The branches within the *Xylaria* species group were densely clustered, reflecting a high degree of genetic similarity among these species during evolution. Notably, *Xylaria* sp. VDL4 exhibited the highest homology with *X. arbuscula*. In this study, phylogenetic analysis of *Xylaria* sp. allowed us to estimate the divergence times and their confidence intervals between *Xylaria* sp. and other species (Fig. 5). The divergence time from *X. arbuscula* B was ~62.8 million years ago, while the divergence from *X. arbuscula* C occurred around 30.2 million years ago. These divergence times not only provide crucial insights into the evolutionary history of these species but also facilitate further exploration of the geological and environmental factors influencing species differentiation.

### Metabolomics analysis

#### Quality control

Pearson correlation analysis was performed on QC samples. The entire experiment was conducted using two scanning modes: the positive ion detection mode (ESI+), which exclusively scans positively charged ions while filtering out negatively charged ions, and the negative ion detection mode (ESI-), which scans only negatively charged ions while filtering out positively charged ions. A higher correlation among QC samples (with | r | closer to 1) indicates greater stability in the detection process and higher data quality. The results demonstrated that sample correlations fluctuated between 0.998 and 1, all of which were close to 1 (Fig. S6).

#### PCA of samples

**Fig. 6.**
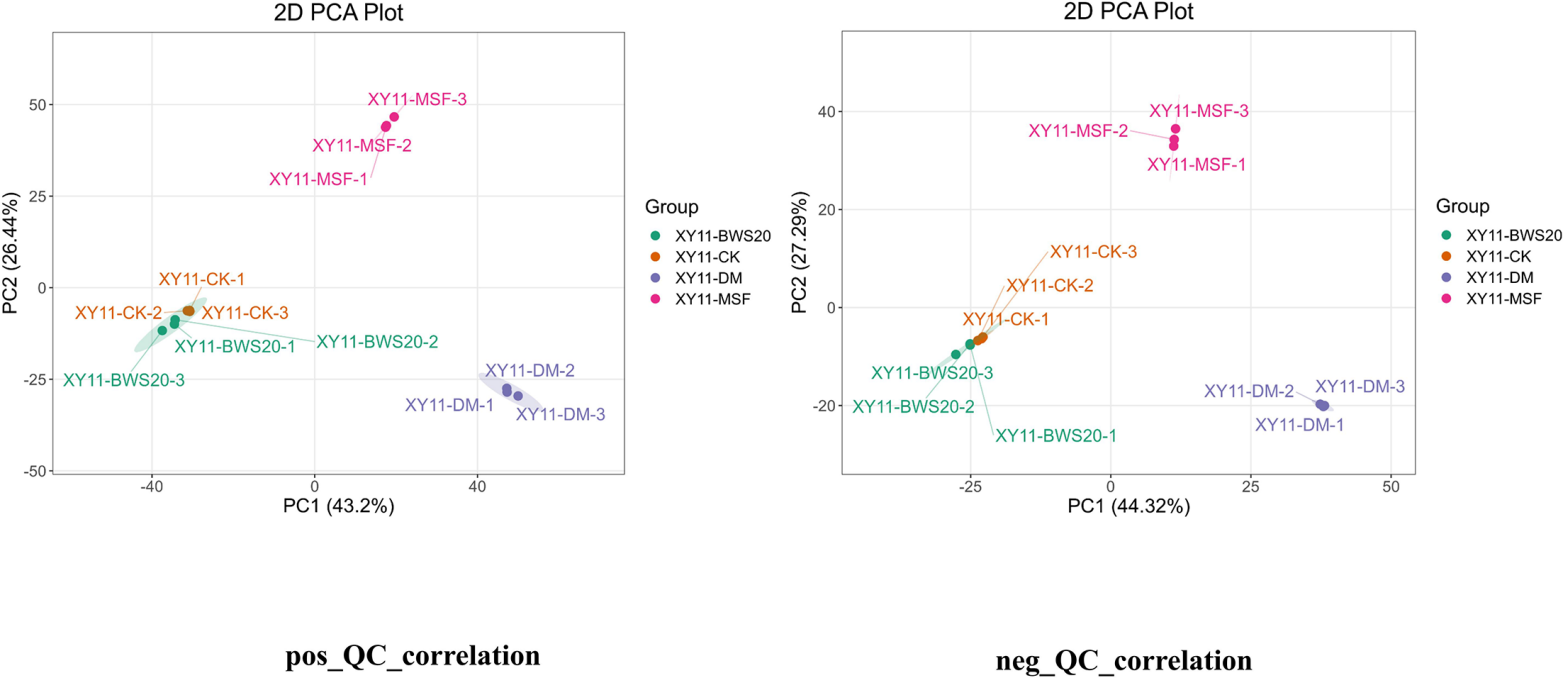
PCA score plot of mass spectrometry data for each sample group vs. quality control samples. Note: PC1 represents the first principal component, and PC2 represents the second principal component, with the percentage indicating the explained variance of the dataset by each principal component. Each point in the plot corresponds to a sample, and samples from the same group are displayed in the same colour, with ‘Group’ indicating the classification. CK, mycelia cultured in yeast malt broth medium; BWS, mycelia cultured in yeast malt broth medium supplemented with valproic acid; DM, mycelia cultured on rice medium; MSF, mycelia cultured on cassava flour medium.

PCA was performed on the samples (including QC samples) to preliminarily assess the overall metabolic differences between groups and the variability within each group. The PCA results revealed distinct clustering trends among the groups, suggesting potential metabolic differences between sample groups [83]. The degree of clustering reflects sample variability – smaller differences among QC samples indicate better methodological stability and higher data quality, which is visually represented by tightly clustered QC sample points in the PCA score plot. As shown in Fig. 6, QC samples (represented by distinct coloured points) exhibited strong clustering in both positive ion (ESI+) and negative ion (ESI-) modes, indicating minimal variability among QC samples. This demonstrates the high stability of the experimental method and the reliability of the acquired data. Using untargeted metabolomics, we investigated the effects of different culture conditions on the secondary metabolites of *Xylaria* spp. PCA revealed overall metabolic shifts between the untreated and treated groups. Notably, three distinct clusters were observed: one for MSF, one for DM and a third group comprising CK and BWS (supplemented with 20 µl valproic acid in yeast malt broth). There was no significant difference between the BWS and CK treatments, indicating that valproic acid did not exert a notable regulatory effect on the accumulation ofsecondary metabolites.

#### Identification of differential metabolites

**Fig. 7.**
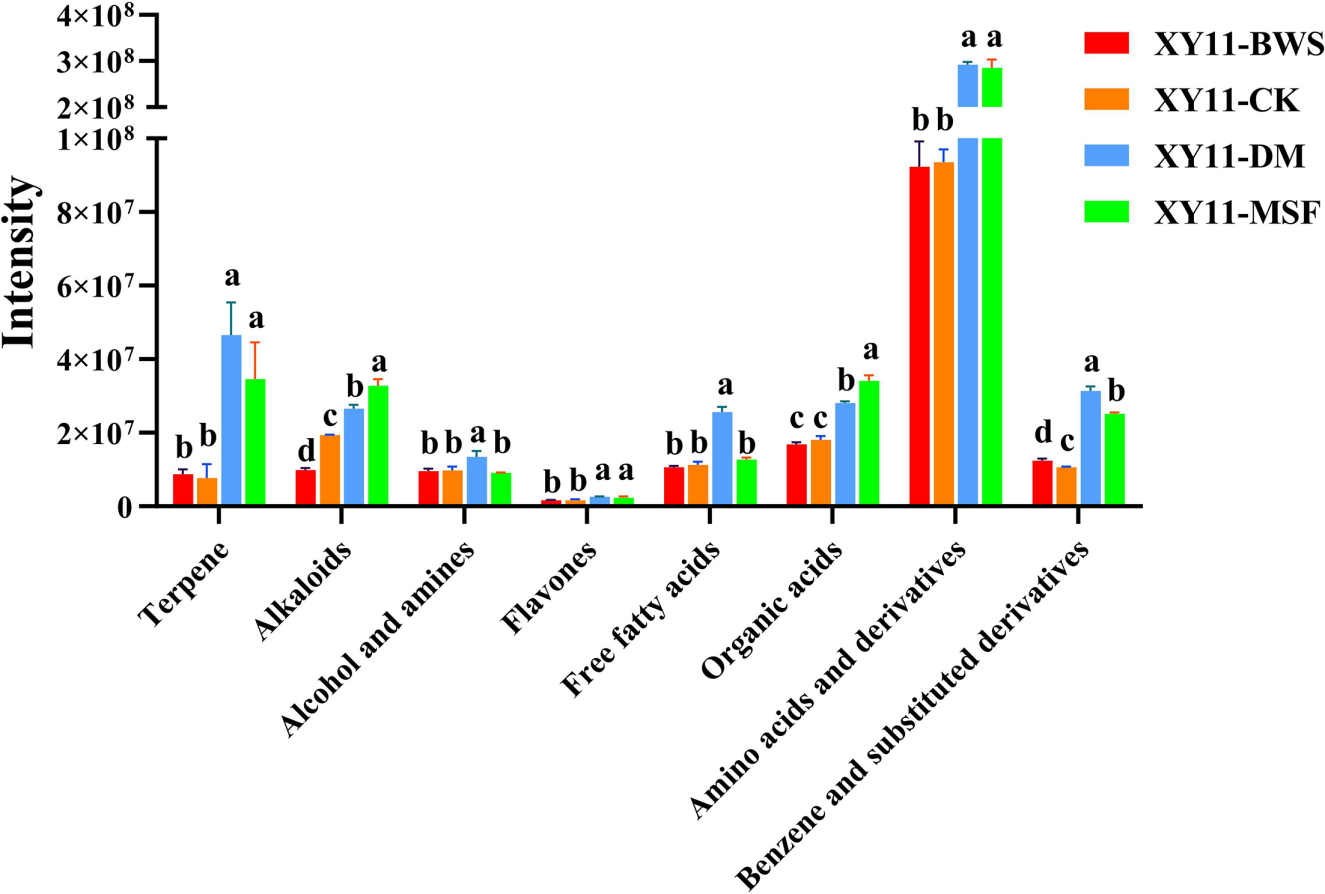
The relative content of secondary metabolites of *Xylaria* sp. VDL4 in different samples. CK, mycelia cultured in yeast malt broth medium; CK, mycelia cultured in yeast malt broth medium; BWS, mycelia cultured in yeast malt broth medium supplemented with valproic acid; DM, mycelia cultured on rice medium; MSF, mycelia cultured on cassava flour medium. The vertical bar represents the standard deviation of the mean (*n*=3), and similar letters inside the same treatment are statistically equivalent at *P* 0.05, based on Tukey’s multiple range test.

The primary research strategy involves comparative analysis between experimental and control groups to detect metabolites present in the samples, obtain quantitative information and identify differentially abundant metabolites with statistical significance across different groups. These metabolites are mapped to metabolic pathways by integrating public databases (KEGG, HMDB and Metlin) with predictive annotation (metDNA), and their directional changes (up- or down-regulation) are displayed on KEGG pathway maps. Differential metabolites are mined by coupling univariate and multivariate analyses tailored to the data structure. Univariate tests comprise hypothesis testing and fold change (FC) analysis; multivariate models include PCA and OPLS-DA. VIP scores from OPLS-DA (≥3 biological replicates) provide an initial filter, which is subsequently refined by univariate *P*-values or false discovery rates (≥2 replicates) and FC thresholds (FC≥2 or ≤0.5). A metabolite is deemed significantly differential when its abundance changes by >2-fold or <0.5-fold between control and experimental groups. In this study, 12 samples were selected and divided into four groups for metabolic research. According to the conditions we set, 1932 different metabolites were screened out in the liquid culture plus valproic acid treatment group, including 816 up-regulated metabolites and 1116 down-regulated metabolites. A total of 2298 different metabolites were detected in the solid culture treatment group. Among them, 790 metabolites were up-regulated and 1508 metabolites were down-regulated. The identified secondary metabolites include terpenoids, alkaloids, phenols and flavonoids. The screened secondary metabolites were analysed by plotting using GraphPad Prism 10.1.2 software. The comparative analysis of the total abundance of each metabolite in the four treatment groups showed that the content of alkaloids increased significantly under the condition of adding cassava flour in solid culture. In rice medium, the contents of four secondary metabolites, such as terpenoids and flavonoids, increased significantly. In addition, the study also found that the content of compounds in solid culture was significantly higher than that in liquid culture (Fig. 7).

### Characterization of *XyTPS* proteins

**Fig. 8.**
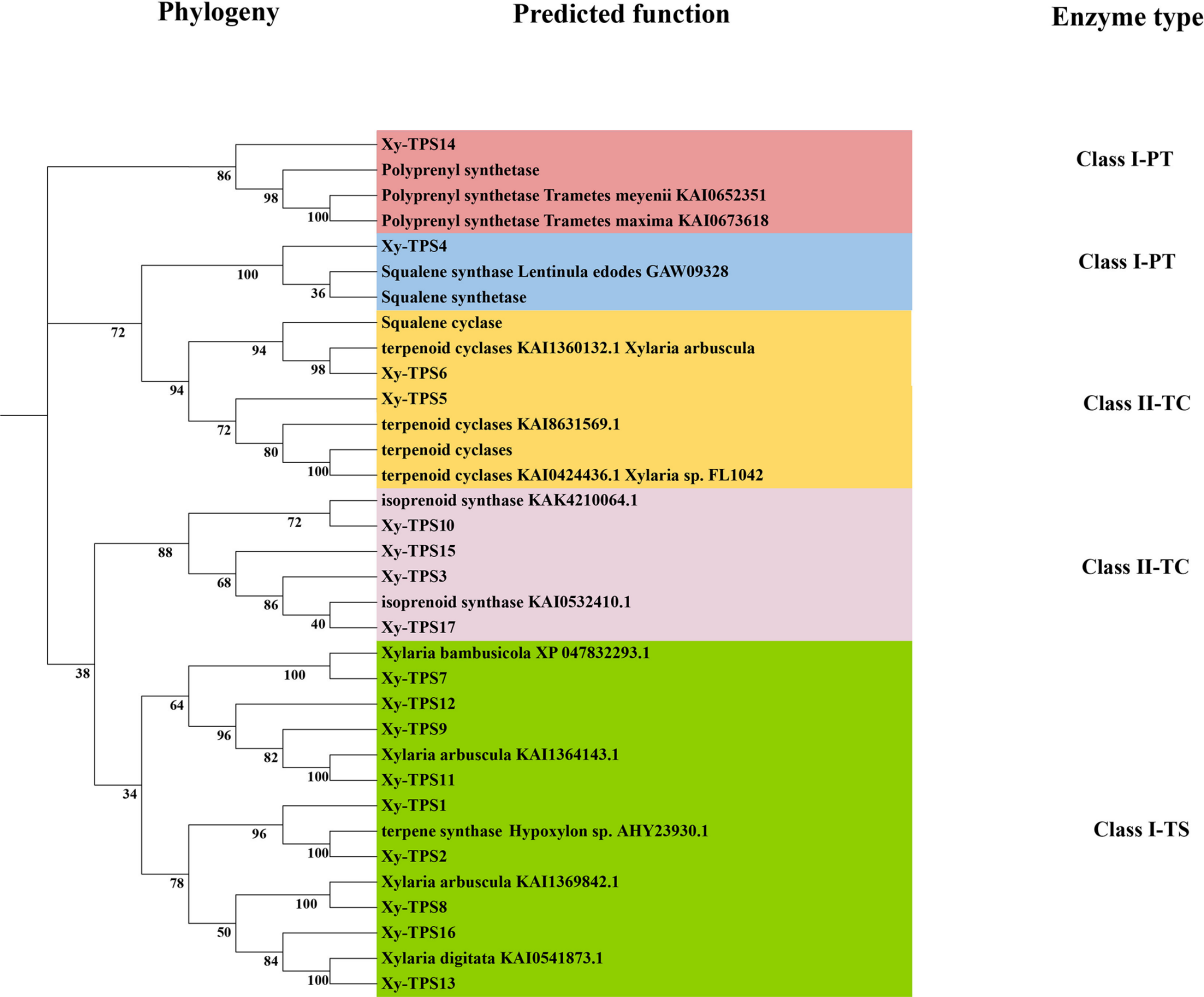
Genomic inventory for terpenoid biosynthesis in *Xylaria* sp. VDL4. Note: *Xy-TPS* is a terpene synthase retrieved from the genome data; the numbers following other species names indicate the sequence accession numbers downloaded from NCBI; the numbers on the branches denote the pairwise similarities of the sequences in the phylogenetic tree.

A total of 17 TPS genes were identified in the genome of *Xylaria* sp. VDL4, comprising 3 terpene cyclase-like 2 genes, 7 terpene synthases, 2 prenyltransferases, 1 squalene synthase (SQS), 2 squalene cyclases, 1 monoterpene synthase and 1 polyprenyl synthetase-like enzyme (Fig. S4). Phylogenetic analysis revealed that *Xy-TPS4* contains the conserved ‘DXXXDD’ motif and clusters with a squalene synthase from *Lentinula edodes* (GAW09328) with 100% homology (Fig. 8), suggesting its potential involvement in triterpenoid biosynthesis. *Xy-TPS5* and *Xy-TPS6* form a clade with terpenoid cyclases from *X. arbuscula* (KAI1360132.1) and *Xylaria* sp. FL1042 (KAI0424436.1), sharing 94% homology, and are likely involved in cyclization reactions to form the cyclic backbone structures of terpenoids. Additionally, nine other TPS genes cluster with proteins from * X. bambusicola* (XP_047832293.1), *X. arbuscula* (KAI1364143.1, KAI1369842.1), *Hypoxylon* sp. (AHY23930.1) and *Xylaria digitata* (KAI0541873.1). These genes group with terpene synthases and may catalyse the polymerization and cyclization of isoprene units to generate diverse terpenoid compounds.

### Phylogenetic tree construction and comparative analysis of PKS

A total of 31 putative PKS genes were identified in the genome of *Xylaria* sp. VDL4 (Table S2), comprising 5 PKS-NRPS hybrids, 9 non-reducing PKSs (NR-PKSs), 7 PR-PKSs and 10 HR-PKSs. To infer their potential biochemical functions, we performed genome-wide prediction across 13 related strains and retrieved 90 orthologous PKS genes. These sequences were phylogenetically classified, and those clustering within the same phylogenetic clade were considered functionally homologous. To visualize the evolutionary relationships among these polyketide synthase genes, a maximum-likelihood phylogenetic tree was constructed (Fig. S8). Branches were colour-coded according to PKS subtypes, clearly demarcating nine functional clades. This visualization enabled the rapid assignment of each VDL4 PKS gene to its cognate functional group, thereby supporting the clade-comparison strategy employed to infer the putative functions of PKS genes in *Xylaria* sp. VDL4. Phylogenetic analysis predicted that *XyPKS3*, *XyPKS12*, *XyPKS15*, *XyPKS17, XyPKS20* and *XyPKS27* cluster within clades containing PKS genes of known function, suggesting similar biosynthetic capabilities. Domain architecture analysis further refined these predictions: *XyPKS12*, *XyPKS15*, *XyPKS17* and *XyPKS27* were identified as NR-PKSs, while *XyPKS3* and *XyPKS15* are PR-PKSs. Specifically, *XyPKS27* shares high structural and domain similarity with YWA1 synthetase and clusters phylogenetically with it, implicating its involvement in YWA1 biosynthesis [84]. Although *XyPKS20* possesses a SAT-KS-AT-DH-ACP-MT-TE domain organization similar to that of *XyPKS27*, phylogenetic analysis places it in a distinct evolutionary branch, leading to the hypothesis that *XyPKS20* may be associated with terpenoid synthesis. *XyPKS17* shows high sequence homology and domain consistency with scytalone synthases, suggesting a potential role in scytalone biosynthesis [85]. *XyPKS3* and *XyPKS15* form a single clade, and given that *XyPKS3* has been linked to flavonoid synthesis, we hypothesize that XyPKS15 may also participate in flavonoid biosynthesis. In contrast, *XyPKS3*, *XyPKS7* and *XyPKS16* did not cluster with any known PKS genes and exhibited unique domain architectures, indicating they may represent novel types of non-reducing PKSs. Collectively, these findings provide important clues for further investigation into the biosynthetic mechanisms of secondary metabolites in *Xylaria* sp. VDL4.

### Analysis of secondary metabolite biosynthesis gene cluster

**Fig. 9.**
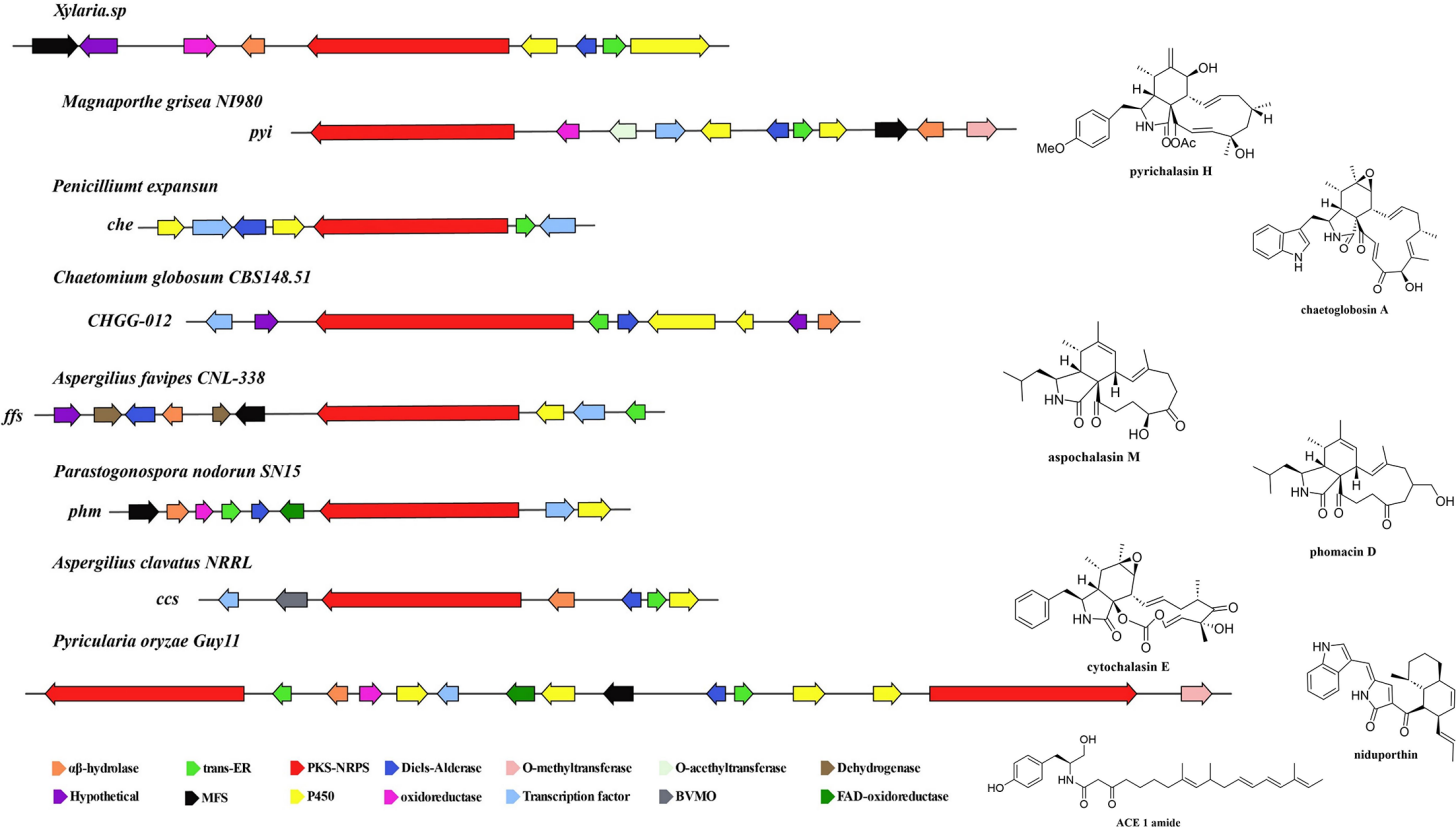
Comparison of biosynthesis of putative cytochalasan BGCs. Note: The source strain of each gene cluster is indicated above; arrows denote different gene names; the chemical structure on the right shows the secondary metabolite synthesized by the corresponding gene cluster.

The results revealed 76 BGCs, including 31 PKS genes, 17 TPS genes and 16 NRPS genes (Table S2 and Table S3). Comparative analysis of PKS-NRPS gene clusters in seven genomes of *Parastogonospora nodorum* SN15, *Penicillium expansum*, *Aspergillus flavipes* CNL-338, *Aspergillus clavatus* NRRL1, *Chaetomium globosum* CBS148.51, *Magnaporthe grisea* NI980 and *Magnaporthe oryzae Guy11* (Table S4) was conducted (Fig. 9). The results indicated that in the seven genomes compared, all contained proteins similar to *XyPKS-NRPS2*, with the PKS-NRPS domain being KS-AT-DH-MT-KR-ACP-C-A-TE, and constructed a phylogenetic tree with five *XyPKS-NRPS* in the genome of *Xylaria* sp. VDL4 (Fig. S9). Phylogenetic analysis showed that *XyPKS-NRPS2* was clustered with these seven PKS-NRPS genes and had high homology. At the same time, there are gene deletions and horizontal gene transfer in each gene cluster, and the differences in the surrounding modified genes confirm the diversity of cytochalasin compounds, revealing the coexistence of high conservation and diversity of biosynthetic pathways. The PKS-NRPS of the comparative genome mainly produces cytochalasin compounds, so it is speculated that the main function of *XyPKS-NRPS2* is related to the synthesis of cytochalasin or its derivatives.

**Fig. 10.**
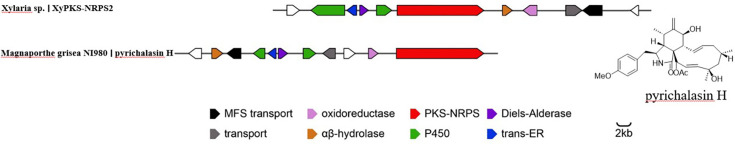
Comparison results of *XyPKS-NRPS2* and pyrichalasin H gene clusters. Note: The source strain of each gene cluster is indicated above; arrows denote different gene names; the chemical structure on the right shows the secondary metabolite synthesized by the corresponding gene cluster.

**Fig. 11.**
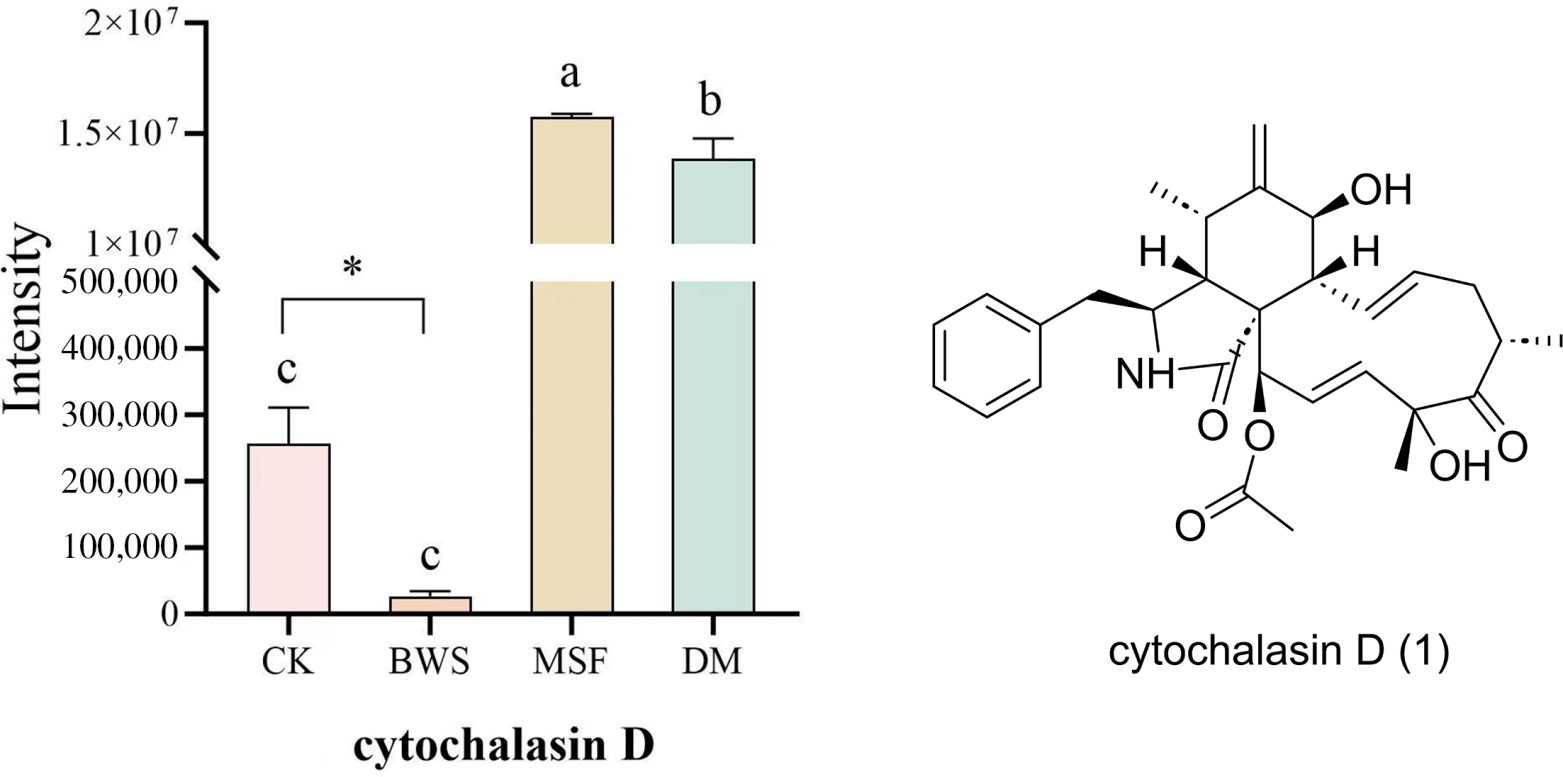
Content differences of cytochalasin D under different culture conditions and compound structure diagrams. Note: The vertical bar represents the standard deviation of the mean (*n*=3), and similar letters inside the same treatment are statistically equivalent at *P* 0.05, based on Tukey’s multiple range test. CK, mycelia cultured in yeast malt broth medium; BWS, mycelia cultured in yeast malt broth medium supplemented with valproic acid; DM, mycelia cultured on rice medium; MSF, mycelia cultured on cassava flour medium. Under solid-state fermentation, cytochalasin D yield was markedly higher than in submerged culture; supplementation with valproic acid caused a sharp drop in its level relative to the CK, indicating that BWS treatment effectively suppresses accumulation of the compound.

**Fig. 12.**
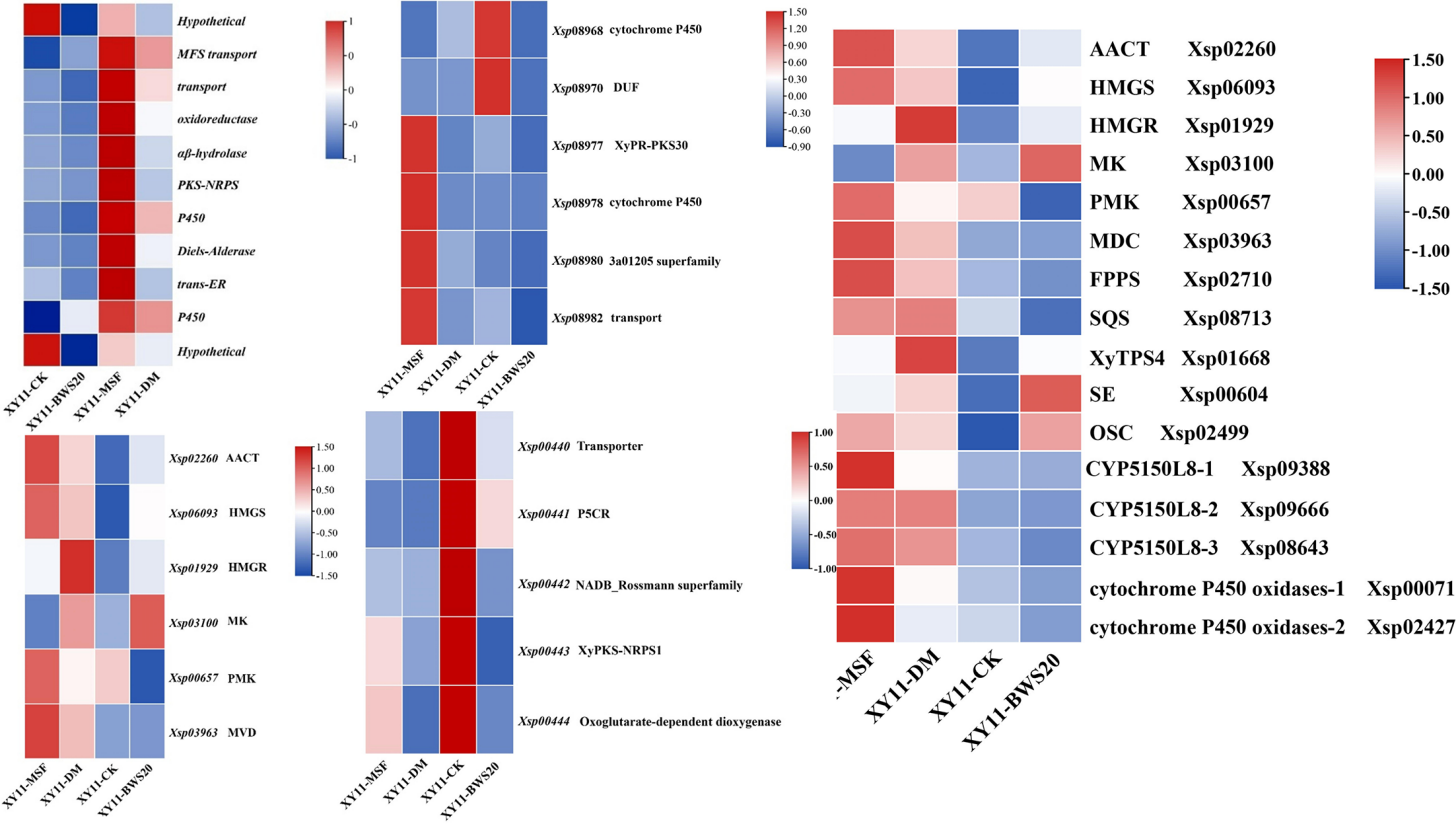
Heat map of the expression levels of enzyme genes in the biosynthetic pathway. Note: IDI, isopentenyl-diphosphate isomerase; GGPPS, geranylgeranyl pyrophosphate synthetase; G10H, geraniol 10-hydroxylase; 10-HGO, 10-HG oxidoreductase; SCS, secologanin synthase; TSB, *β*-subunit of tryptophan synthase; TDC, tryptophan decarboxylase; SQS, squalene synthase; AACT, acetyl-CoA C-acetyltransferase; HMGS, 3-hydroxymethyl-3-methylglutaryl-CoA lyase; HMGR, hydroxymethylglutaryl-CoA reductase; MK, mevalonate kinase; PMK, phosphomevalonate kinase; MVD, diphosphomevalonate decarboxylase; FPPS, farnesyl pyrophosphate synthase; SE, squalene epoxidase; OSC, 2,3-oxidosqualene-lanosterol cyclase; STR, strictosidine synthase; CK, mycelia cultured in yeast malt broth medium; BWS, mycelia cultured in yeast malt broth medium supplemented with valproic acid; DM, mycelia cultured on rice medium; MSF, mycelia cultured on cassava flour medium.

The comparative genomics and phylogenetic tree analysis indicated that *XyPKS-NRPS2* had the highest homology with the PKS-NRPS homologues in the genome of *Magnaporthe grisea* NI980. Moreover, the BGC *pyi* for the synthesis of pyrichalasin H in *Magnaporthe grisea* NI980 was analysed by bioinformatics. It was found that in addition to the PKS-NRPS gene *pyiS*, the pyi cluster contained a trans-enoyl reductase (trans-ER; *pyiC*), two P450 monooxygenases (*pyiD* and *pyiG*), an *α*/*β*-hydrolase (*pyiE*), a transcription factor (*pyiR*), an MFS transporter (*pyiT*), an oxidoreductase (*pyiH*), an O-methyl-transferase (*pyiA*), an O-acetyl-transferase (*pyiB*) and a putative Diels–Alder enzyme (putative Diels–Alderase, *pyiF*), which was considered to be involved in the intramolecular [4+2] cycloaddition reaction. The clustering analysis results showed that the homology of *XyPKS-NRPS23*, *XyPKS-NRPS25* and *XyPKS-NRPS31* with the PKS genes related to the synthesis of cellobiose was relatively low, and this might be related to the biosynthesis of other compounds (Figure S9). The PKS-NRPS domain of *XyPKS-NRPS2* is consistent with the PKS-NRPS domain of pyrichalasin H, and the similarity of the gene cluster is 81%. The gene cluster of *XyPKS-NRPS2* gene contains genes that synthesize cytochalasans: MFS (Xsp01198), oxidoreductase (Xsp01200), *α*/*β* hydrolase (Xsp01201), PKS-NRPS (Xsp01202), cytochrome P450 (Xsp01203), Diels–Alderase (Xsp01204) and trans-enoyl reductase (Xsp01205) (Fig. 10). In this study, cytochalasin D was successfully detected by metabolomics analysis, suggesting that *XyPKS-NRPS2* could catalyse the synthesis of cytochalasin D. The results showed that the content of cytochalasin D was significantly different under different culture conditions (Fig. 11). Further analysis showed that the expression level of cytochalasin D was significantly higher than that of other conditions under the condition of adding cassava flour. This result is highly consistent with the expression pattern of heatmap data (Fig. 12), so it has high reliability. This high degree of consistency indicates that there is a close relationship between changes in gene expression and changes in cytochalasin D content. The up-regulation or down-regulation of gene expression may directly affect the biosynthesis process of cytochalasin D, which in turn leads to the difference in its content under different culture conditions. Under solid-state fermentation, cytochalasin D yield was markedly higher than in submerged culture; supplementation with valproic acid caused a sharp drop in its level relative to the CK, indicating that BWS treatment effectively suppresses accumulation of the compound (Fig. 11). Transcriptomic analysis revealed that the PKS-NRPS2 gene governing cytochalasin D biosynthesis, together with the adjacent cytochrome P450 tailoring genes, was significantly up-regulated in solid-state vs. liquid cultures, and their expression in the BWS group was markedly lower than in the CK group (Fig. 12). The metabolomic profile closely paralleled this transcriptional pattern, corroborating the reliability of the data. Therefore, alterations in culture conditions could substantially influence cytochalasin D yield through modulating the silenced expression of the BGC.

**Fig. 13.**
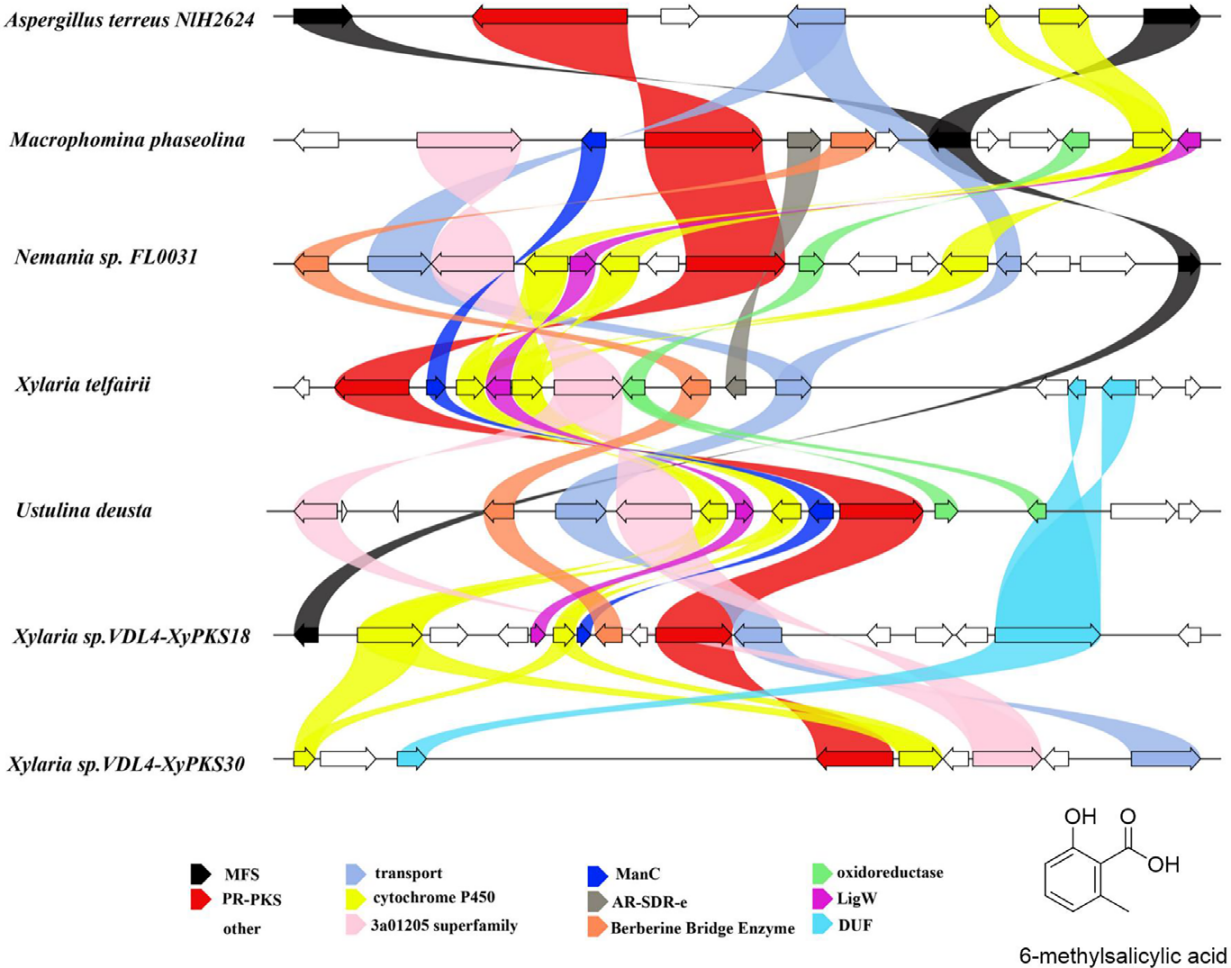
Comparison of biosynthesis of putative 6MSA BGCs. Note: The source strain of each gene cluster is indicated above; arrows denote different gene names; the chemical structure on the right shows the secondary metabolite synthesized by the corresponding gene cluster.

In the five genomes of *Aspergillus terreus* NlH2624, *Penicillium griseofulvum*, *Macrophomina phaseolina*, *Nemania* sp. FL0031 and *X. telfairii* (Table S5), each contains a PR-PKS with a KS-AT-DH-KR-ACP domain architecture associated with 6-methylsalicylic acid (6MSA) biosynthesis, exhibiting identical domain organization to *XyPKS30*. Phylogenetic analysis was performed by clustering these five known PR-PKSs with those from *Xylaria* sp. VDL4 (Fig. S10), revealing that *XyPKS30* and *XyPKS18* share over 80% homology with PKS genes involved in 6MSA synthesis. Based on phylogenetic tree analysis and gene cluster alignment. Furthermore, *P. griseofulvum*, *P. expansum* PatK, *Macrophomina phaseolina*, *Nemania* sp. FL0031 and *X. telfairii* possess identical modifying genes to those in *Xylaria* sp. VDL4, including Berberine Bridge Enzyme, cytochrome P450, DUF, 5-carboxyvanillate decarboxylase (LigW) and MFS (Fig. 13). These findings suggest that *XyPKS30* likely catalyses the synthesis of 6MSA [86,87]. It is noteworthy that 6MSA is generally not the final product in metabolic pathways, but rather serves as a key precursor for structurally diverse secondary metabolites. The formation of its final product may be regulated by the expression of post-modification genes, and this finding provides new research directions for our subsequent validation of gene functions. Under solid culture conditions, the overall expression levels of core genes responsible for 6-methylsalicylic acid synthesis in MSF were significantly higher than those under other treatments; only DUF family protein genes and one cytochrome P450 gene exhibited higher expression under CK conditions (Fig. 12).

**Fig. 14.**
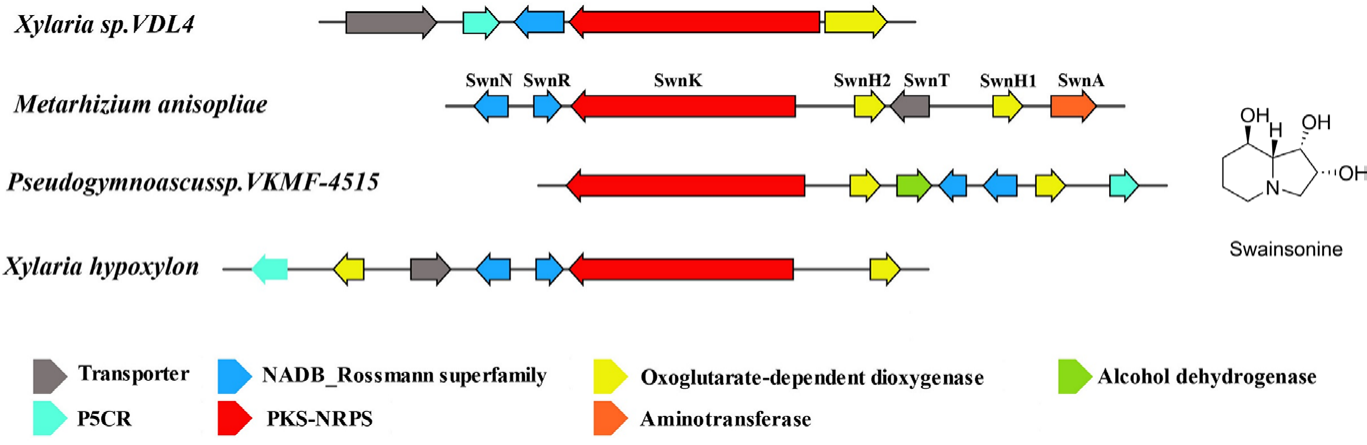
Comparison of biosynthesis of putative swainsonine BGCs.

Swainsonine is a type of indolizidine alkaloid synthesized by fungi and can cause mad cow disease in mammals. It is also a potential anti-cancer drug [88]. By comparing the genomes of *Metarhizium anisopliae*, *Pseudogymnoascus* sp. VKMF-4515h and * X. hypoxylon*, a gene cluster related to the biosynthesis of swainsonine, ‘SWN’, was found to contain genes such as swnA, swnR, swnN, swnH1, swnH2 and swnK [89]. Among them, the swnK gene is a multifunctional enzyme-coding gene, consisting of multiple functional domains, such as adenylate lyase (adenylylation, A), *β*-ketoacyl synthase (KS), acyltransferase (AT), reductase (SDR) and thioester reductase (SDRel) (Fig. 14). Luo *et al*. proved that the swnK gene is the core gene for swainsonine production [90]. Through the analysis of gene cluster comparison, it can be known that the multifunctional enzyme encoding gene of *XyPKS-NRPS1* is consistent with Swnk. The *Xylaria* sp. VDL4 BGC has a pyrroline-5-carboxylate reductase-like (P5CR) gene that is absent from the swn BGC from *Metarhizium robertsii* but is present in the homologous BGC from *Pseudogymnoascus* sp. VKM F-4515 (Fig. 14). Screening of the culture crude extracts from *Xylaria* sp. VDL4 for swainsonine content did not indicate the presence of such molecules, but the P5CR-like protein might be involved in the alteration of the structure, preventing identification of the pathway product. cblaster homology searches using the SwnK, SwnH1/2, SwnR and SwnN protein sequences from *Xylaria* sp. VDL4 demonstrated that homologous BGCs are present in other *Xylaria* sp*.* and related fungi (e.g. *Rosellinia necatrix*, * X. grammica*, *Xylaria multiplex*), indicating that the swainsonine-like BGC is common within the *Xylariaceae* [91,92]. Moreover, by constructing a phylogenetic tree, it can be found that the homology degree between *XyPKS-NRPS1* and Swnk is up to 98%. Therefore, it is speculated that *XyPKS-NRPS1* can catalyse the synthesis of swainsonine. The differences in the surrounding modification genes may lead to significant differences in the biosynthesis pathways of swainsonine among different fungi [93].

Through the analysis of the genome of *Xylaria* sp. VDL4, we identified the TPS gene and constructed a phylogenetic tree. The results showed that *Xy-TPS4* clustered with squalene synthase into one branch, with a homology of 100% (Fig. 8). It is known that SQS is a key enzyme in the biosynthetic pathway of triterpenoids, catalysing the condensation of two molecules of farnesyl pyrophosphate to form squalene. Squalene synthase is a precursor for the synthesis of triterpenoids and is widely involved in the biosynthesis of secondary metabolites, such as sterols and triterpenoid saponins. Ganoderic acid Mf (GA-Mf), a lanostane-type triterpenoid from *Ganoderma lucidum* whose complete biosynthetic pathway remains elusive, is presumed to originate from acetyl-CoA via the mevalonate pathway to 2,3-oxidosqualene, the common precursor for sterols and lanostane-type triterpenoids. Previous studies have established that the cytochrome P450 mono-oxygenases CYP5150L8 (gene model GL24883) and CYP512U6 (GL31761) participate in *Ganoderma* triterpenoid biosynthesis: CYP512U6 hydroxylates C-23 of ganoderic acids DM and TR to yield hainanic acid A and ganoderic acid Jc [94], respectively, whereas CYP5150L8 catalyses C-26 oxidation of lanosterol to produce GA-HLDOA [95]. The structural signature of GA-Mf – namely, a C-26 carboxylic acid, a C-3*β*-hydroxyl group and a side-chain double bond – precisely matches the oxidation pattern of HLDOA/DHLDOA. Therefore, it is speculated that its biosynthesis is first catalysed by CYP5150L8 for C-26 carboxylation to produce HLDOA, followed by further oxidation at C-28 or the side chain by other P450 enzymes, and finally completed by short-chain dehydrogenase/reductase or acyltransferase with the unique modifications of Mf. In this study, it was found that the gene expression patterns of the enzymes in the MVA pathway and the CYP5150L8 enzyme are largely consistent with the metabolomic data (Figs 14 and 17). Therefore, through multi-omics data analysis, we speculated that *XyTPS4* in *Xylaria* sp. VDL4 might be involved in the biosynthesis of ganoderic acid Mf.

**Fig. 15.**
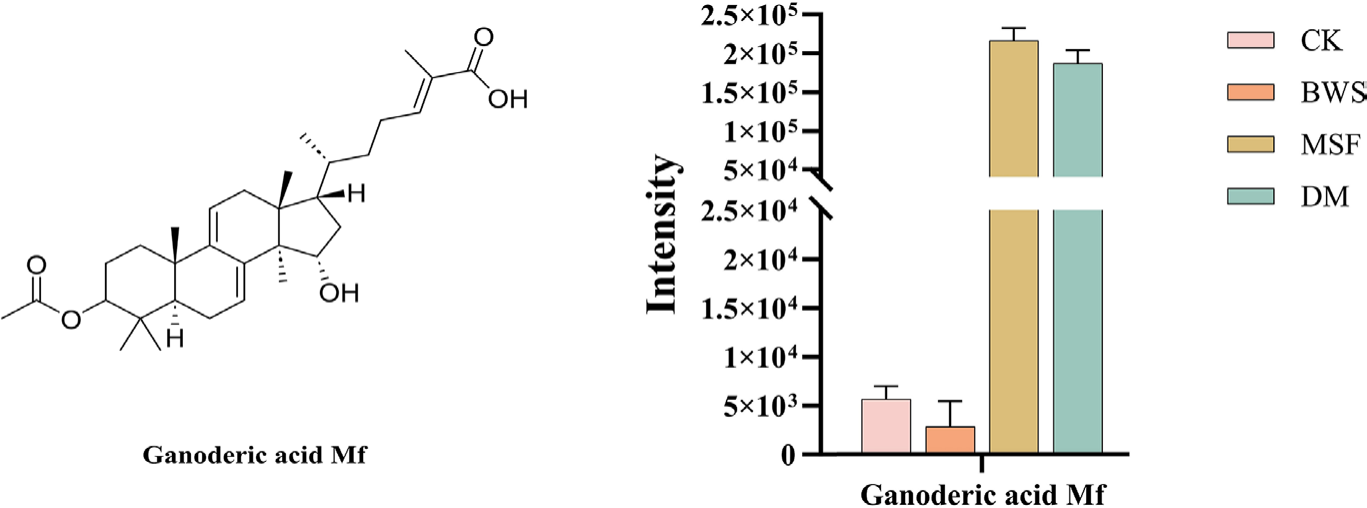
The content differences and compound structures of ganoderic acid Mf under different culture conditions. Note: The vertical bar represents the standard deviation of the mean (*n*=3). CK, mycelia cultured in yeast malt broth medium; BWS, mycelia cultured in yeast malt broth medium supplemented with valproic acid; DM, mycelia cultured on rice medium; MSF, mycelia cultured on cassava flour medium. Under solid-state fermentation, ganoderic acid Mf yield was markedly higher than in submerged culture.

**Fig. 16.**
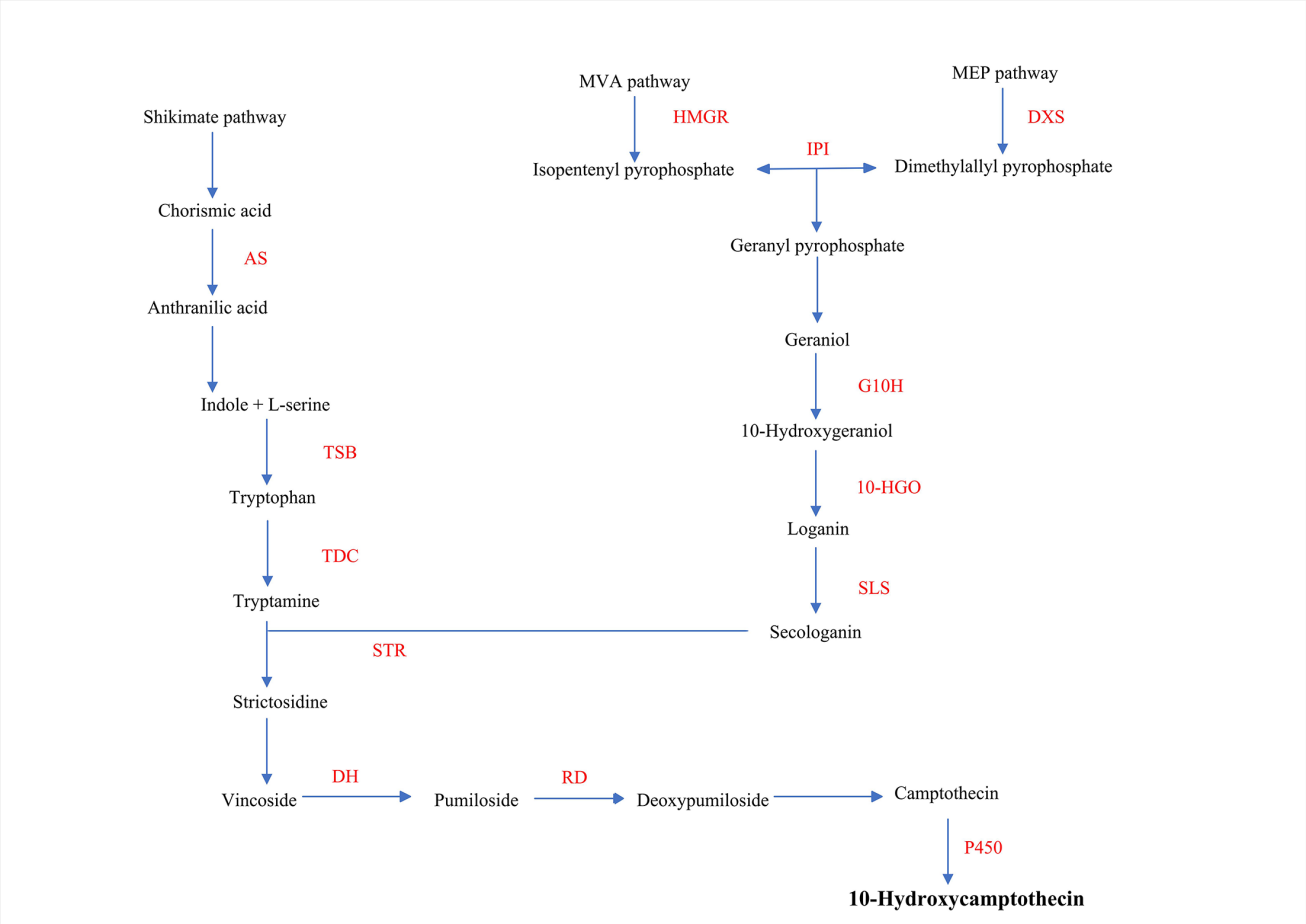
The known biosynthetic flowchart of 10-hydroxycamptothecin. Note: AS, anthranilate synthase; TSB, *β*-subunit of tryptophan synthase; TDC, tryptophan decarboxylase; STR, strictosidine synthase; HMGR, hydroxymethylglutaryl-CoA reductase; IPI, Isopentenyl-diphosphate isomerase; DXS, 1-deoxy-d-xylulose-5-phosphate synthase; G10H, geraniol 10-hydroxylase; 10-HGO, 10-HG oxidoreductase; SLS, secologanic acid synthase.

**Fig. 17.**
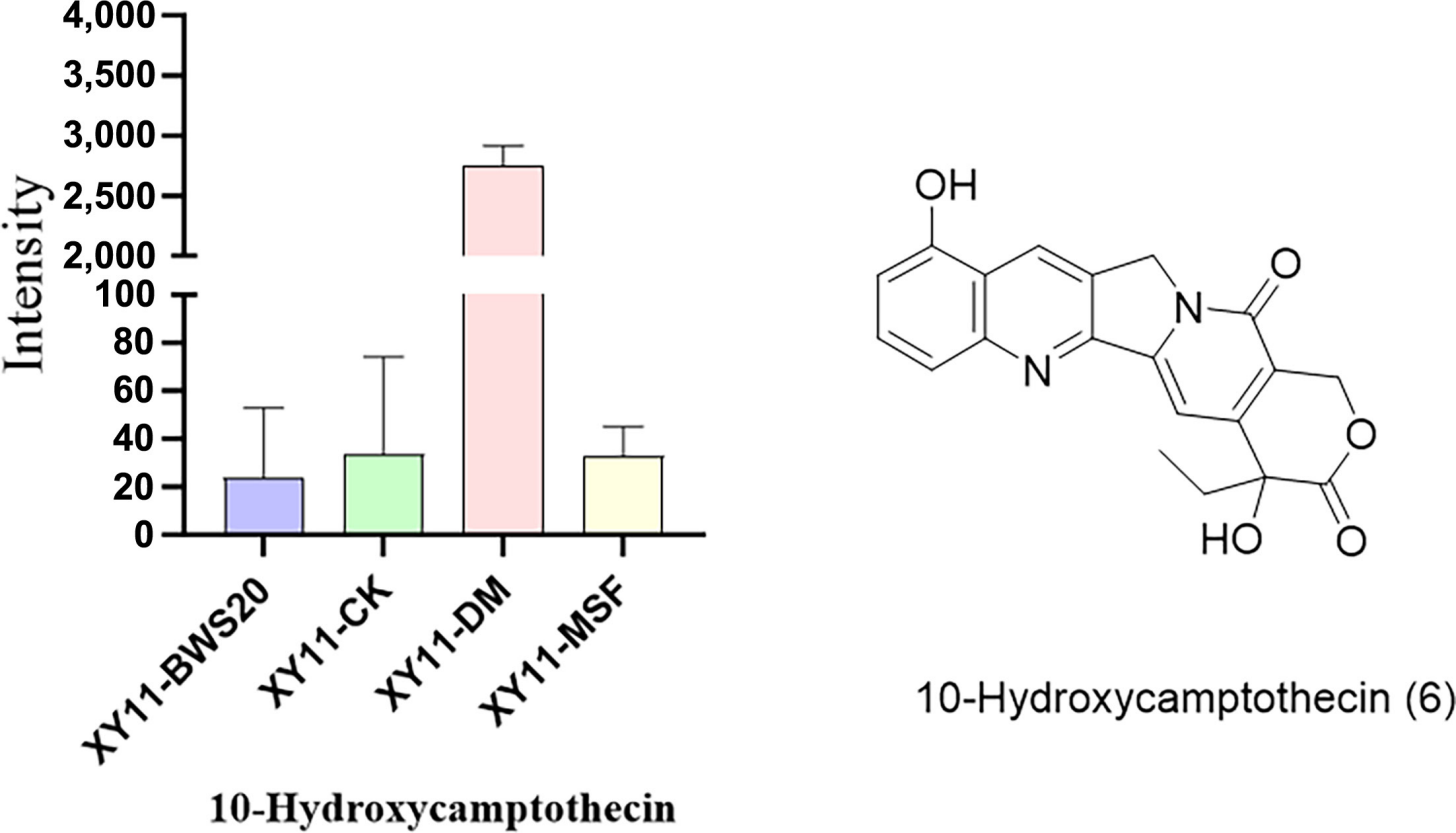
The content differences and compound structures of 10-hydroxycamptothecin under different culture conditions. Note: The vertical bar represents the standard deviation of the mean (*n*=3). CK, mycelia cultured in yeast malt broth medium; BWS, mycelia cultured in yeast malt broth medium supplemented with valproic acid; DM, mycelia cultured on rice medium; MSF, mycelia cultured on cassava flour medium. Under MSF culture conditions, the yield of 10-hydroxycamptothecin was significantly higher than under all other fermentation regimes.

From a biosynthetic perspective, 10-hydroxycamptothecin belongs to the MIAs. Analysis of the TPS gene in *Xylaria* sp. VDL4 suggests that *Xy-TPS11* participates in the biosynthesis of 10-hydroxycamptothecin using geranylgeranyl pyrophosphate synthetase (GGPPS) as a precursor. The biosynthesis of terpenoids primarily occurs via the MVA pathway, whereas indole alkaloid synthesis predominantly relies on the shikimate pathway. Tryptophan decarboxylase (TDC) converts tryptophan into tryptamine, and STR catalyses the condensation of tryptamine and secologanin to form strictosidine, a key precursor compound for terpenoid indole alkaloids [39]. Strictosidine serves as a critical intermediate in the biosynthesis of CPT-type indole alkaloids [40]. Hydroxylation at the C-10 position of camptothecin is a pivotal step in the synthesis of 10-hydroxycamptothecin (Fig. 16), a reaction that profoundly influences the bioactivity and structural diversity of camptothecin derivatives. Metabolomic profiling revealed that the content of 10-hydroxycamptothecin in DM was significantly higher than in MSF, CK and BWS cultures (Fig. 17), whereas no obvious differences were observed among MSF, CK and BWS. Transcriptomic analysis showed that the expression levels of the core genes involved in 10-hydroxycamptothecin biosynthesis – TSB, TDC and STR – as well as the genes of the MVA pathway were all elevated in DM compared with the other media (Fig. 12).

### Gene expression analysis of *XyPKS* and *XyTPS* under different cultivation conditions

Transcriptomic sequencing analysis was performed on the mycelia of *Xylaria* sp. VDL4 under different culture conditions. Gene expression data were collected by retrieving FPKM values based on gene IDs. An interactive heatmap of PKS and TPS gene expression (Fig. S11) was generated using TBtools software to visually represent differential gene expression across cultivation conditions. The analysis revealed that under solid culture conditions (DM and MSF), the expression levels of *XyPKS2*, *XyPKS6*, *XyPKS7*, *XyPKS10*, *XyPKS26*, *XyPKS30*, *Xy-TPS3*, *Xy-TPS10*, *Xy-TPS15* and *Xy-TPS17* were significantly higher than those under liquid culture conditions (MY-CK and MY-BWS). In contrast, *XyPKS3*, *XyPKS21*, *XyPKS25*, *Xy-TPS7* and *Xy-TPS8* exhibited more active expression in liquid cultures, which may be associated with specific biological processes or metabolic pathways in the liquid environment.

### Identification and analysis of transcription factors in *Xylaria* sp. VDL4

**Fig. 18.**
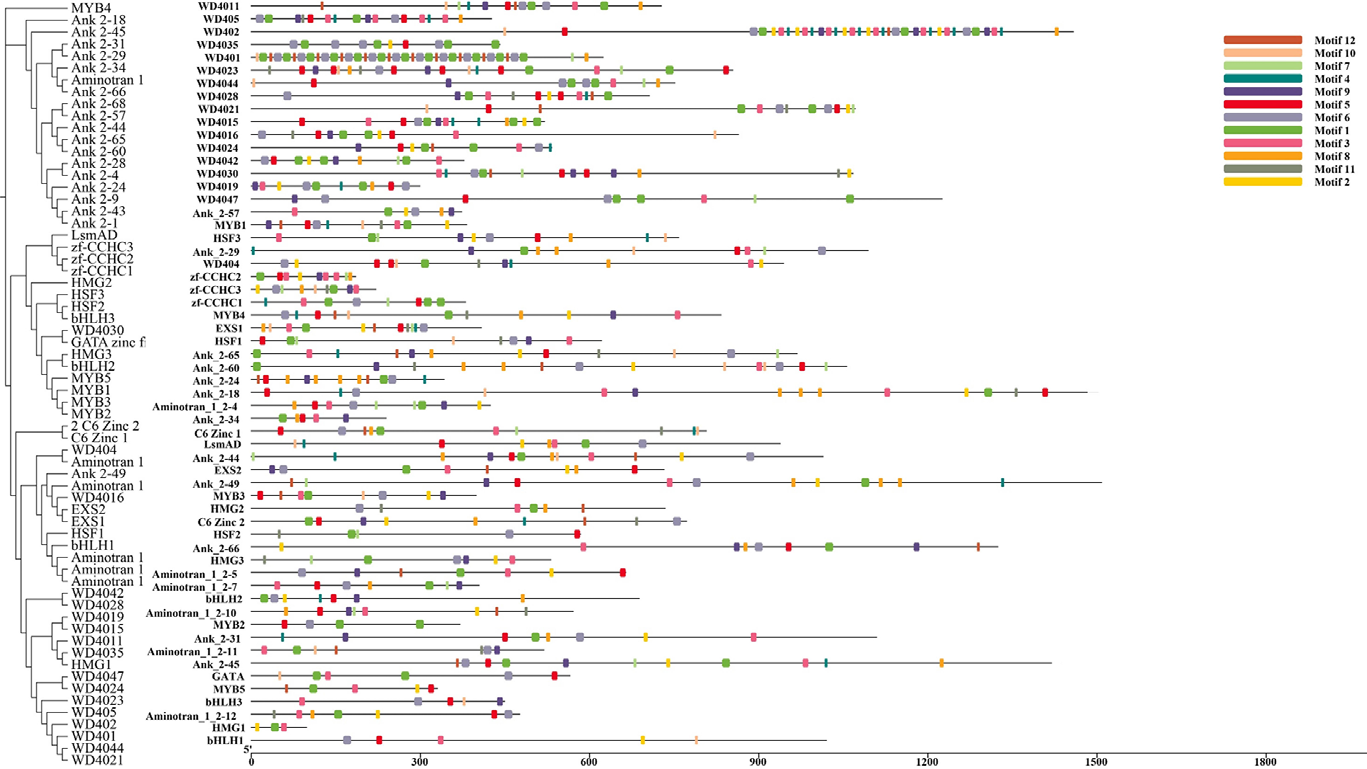
Structural characterization of ten transcription factor families of *Xylaria* sp. VDL4. From left to right: phylogenetic tree of proteins and conserved motif analysis.

After comparative analysis, a total of 171 TF sequences were identified in the genome of *Xylaria* sp. VDL4. These sequences specifically include MYB (5), bHLH (3), C2H2-zf (3), WD40 (50), ARD (1), SRF (1), CBFB (1), CBFD (1), ANK2 (72), zf-CCHC (3), HSF (3), HMG (3), C6 zinc (2), GATA (1), TFIIB (2), HD (2), FOX (3), AAT (12), Trf (1) and EXS (2). A total of 142 TF sequences related to secondary metabolite biosynthesis were identified, namely MYB (5), bHLH (3), WD40 (50), ANK2 (72), zf-CCHC (3), HSF (3), HMG (3), C6 zinc (2), GATA (1) and EXS (2). These sequences were named *XyMYB1* to *XyEXS2*. The phylogenetic trees of these ten TF families were constructed using MEGAX64 software. A total of 12 memes were identified through meme analysis. Memes were generally similar among members of the same family (Fig. 18). Members related to phylogenetic analysis usually have similar motifs. The conserved motifs of transcription factor proteins in different families vary in type, quantity and distribution. These differences are consistent with the results of phylogenetic analysis. It is speculated that different types of differences are related to multiple specific functions in the family. The protein conserved structure was analysed and found that all transcription factors contain their typical conserved domains. The protein domains of the same subgroup have a high degree of similarity.

### Gene expression analysis of *XyTFs* under different cultivation conditions

Selected transcription factors associated with secondary metabolite biosynthesis in *Xylaria* sp. VDL4 were analysed using TBtools software to generate an interactive heatmap, providing a visual representation of gene expression differences under varying culture conditions. Distinct expression patterns of transcription factors were observed across these conditions. Notably, *XyWD40-5*, *XyWD40-30*, *XyWD40-35*, *XyWD40-44*, *XyAnk-2–18*, *XyAnk-2–24*, *XyAnk-2–65*, *XyAnk-2–68*, *XyC6 Zinc2*, *XyHSF3*, *XyAminotran-1-2-4*, *XyAminotran-1-2-5* and *XyAminotran-1-2-11* exhibited higher transcriptional activity under solid culture conditions. In contrast, *XyWD40-15*, *XyWD40-19*, *XyAnk-2–1*, *XyMYB3*, *XyMYB5*, *XybHLH2*, *XyHMG1* and *Xyzf-CCHC1* showed more pronounced expression in liquid culture (Fig. S12). These differential expression patterns may reflect the involvement of these transcription factors in specific biological processes unique to each cultivation method.

### Prediction of *XyTFs*-binding sites in the promoter regions of the *XyPKS* and *XyTPS* genes

**Fig. 19.**
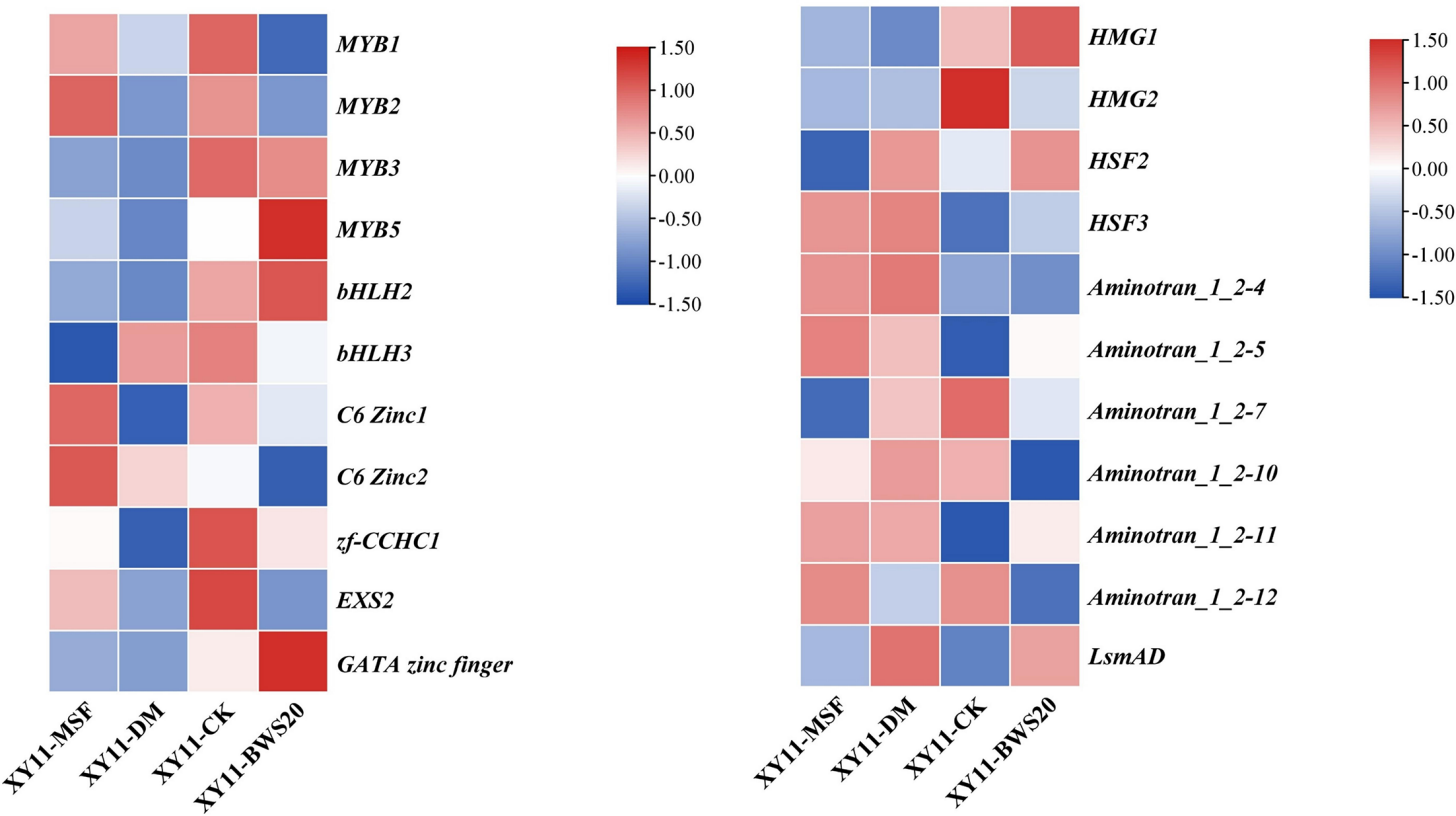
Interactive heatmap of gene expression for (**a**) *XyPKS*, (**b**) *XyTPS* and co-expressed *XyTFs* under varying cultivation conditions. CK, mycelia cultured in yeast malt broth medium; BWS, mycelia cultured in yeast malt broth medium supplemented with valproic acid; DM, mycelia cultured on rice medium; MSF, mycelia cultured on cassava flour medium.

Through comprehensive comparative analysis of the expression patterns of *XyPKS*, *XyTPS* and transcription factors under different culture conditions, the results revealed that under solid culture conditions, the transcription factor *XyMYB2* exhibited consistently high expression levels with *XyPKS18*, while *XyMYB3*, *XyHMG1* and *XybHLH2* showed similar co-expression with *XyPKS25*. Additionally, *XyMYB5* demonstrated synchronized high expression with *Xy-TPS7*, and *XyAnk*_*2–29* correlated with *XyPKS13*. Under liquid culture conditions, *XyMYB1* and *Xy-TPS9*, *XybHLH3* and *Xy-TPS11*, *XyWD40-23* and *Xy-TPS16*, *XyHMG2* and *XyPKS12*, *XyAnk_2–60* and *XyEXS2* and *Xy-TPS2* genes showed similar high expression patterns (Fig. 19). Based on these findings, we hypothesize that these transcription factors may regulate *XyPKS* and *XyTPS* genes. To test this hypothesis, the promoter regions (2,000 bp) of *XyPKS* and *XyTPS* genes were identified using TBtools software, leveraging the whole-genome data of *Xylaria* sp. VDL4.

Using the JASPAR database, the transcription factor binding sites in the promoter region of the *XyPKS* and *XyTPS* genes were analysed in detail, and several potential binding sites with high relative scores were found. These sites may be involved in the regulation of the *XyPKS* and *XyTPS* genes. The specific results are as follows: the binding site of the *XyHMG2* transcription factor in the positive chain of the *XyPKS12* gene promoter region from 1626 to 1631 is ‘tctaga’, which shows a high degree of matching, indicating that this site may be related to the functional regulation of the *XyPKS12* gene. The binding site ‘agccctat’ of *XyMYB2* transcription factor in the positive strand 2305 to 2312 in the promoter region of *XyPKS18* gene has a high relative score of 0.9517559, indicating that this site may be related to the functional regulation of *XyPKS18* gene. The transcription factors *XyMYB3*, *XybHLH2* and *XyHMG1* also show high matching with the promoter region of the *XyPKS25* gene. The binding sites of the MYB3 transcription factor are ‘tacccat’, those of *XybHLH2* transcription factor are ‘gcatgggaa’ and those of *XyHMG1* transcription factor are ‘gaacgtgg’, all of which are highly matched with the promoter region of the *XyPKS25* gene. The binding site of the *XyMYB5* transcription factor for the positive strand 926 to 832 of the promoter region of the terpene synthase *Xy-TPS7* gene is ‘aacccat’, with a high relative score of 0.9939835, indicating that this site is related to the functional regulation of the *Xy-TPS7* gene. The binding site of the *XyMYB1* transcription factor for the positive strand 351 to 358 of the promoter region of the *Xy-TPS9* gene is ‘aaccctac’, showing high matching. The binding site of the *XybHLH3* transcription factor for the promoter region of the terpene synthase *Xy-TPS11* gene is ‘gaacgtgg’ (Table 3). In conclusion, these transcription factors may bind to the DNA sequences of the promoter regions of the corresponding *XyPKS* or *XyTPS* genes, thereby activating the expression of the *XyPKS* or *XyTPS* genes.

**Table 3.** Binding sites of co-expressed *XyTFs* in the promoter regions of *XyPKS* and *XyTPS*

Matrix ID	Score	Relative score	Sequence ID	Start	End	Strand	Predicted sequence
HMG2	10.989888	1	PKS12(Xsp03956)	1,626	1,631	−	tctaga
MYB2	9.973817	0.9517559	PKS18(Xsp06248)	2,305	2,312	+	agccctat
MYB3	11.274977	0.99782276	PKS25(Xsp08052)	641	647	−	tacccat
bHLH2	12.293192	0.9318406	PKS25(Xsp08052)	940	948	−	gcatgggaa
HMG1	11.583604	1	PKS25(Xsp08052)	760	766	−	ctctaga
MYB5	11.101326	0.9939835	TPS-7(Xsp04341)	826	832	+	aacccat
MYB1	10.485749	0.9690819	TPS-9(Xsp06434)	351	358	−	aaccctac
bHLH3	9.680169	0.93297195	TPS-11(Xsp07241)	841	848	−	gaacgtgg

### Quantitative real-time PCR analysis

**Fig. 20.**
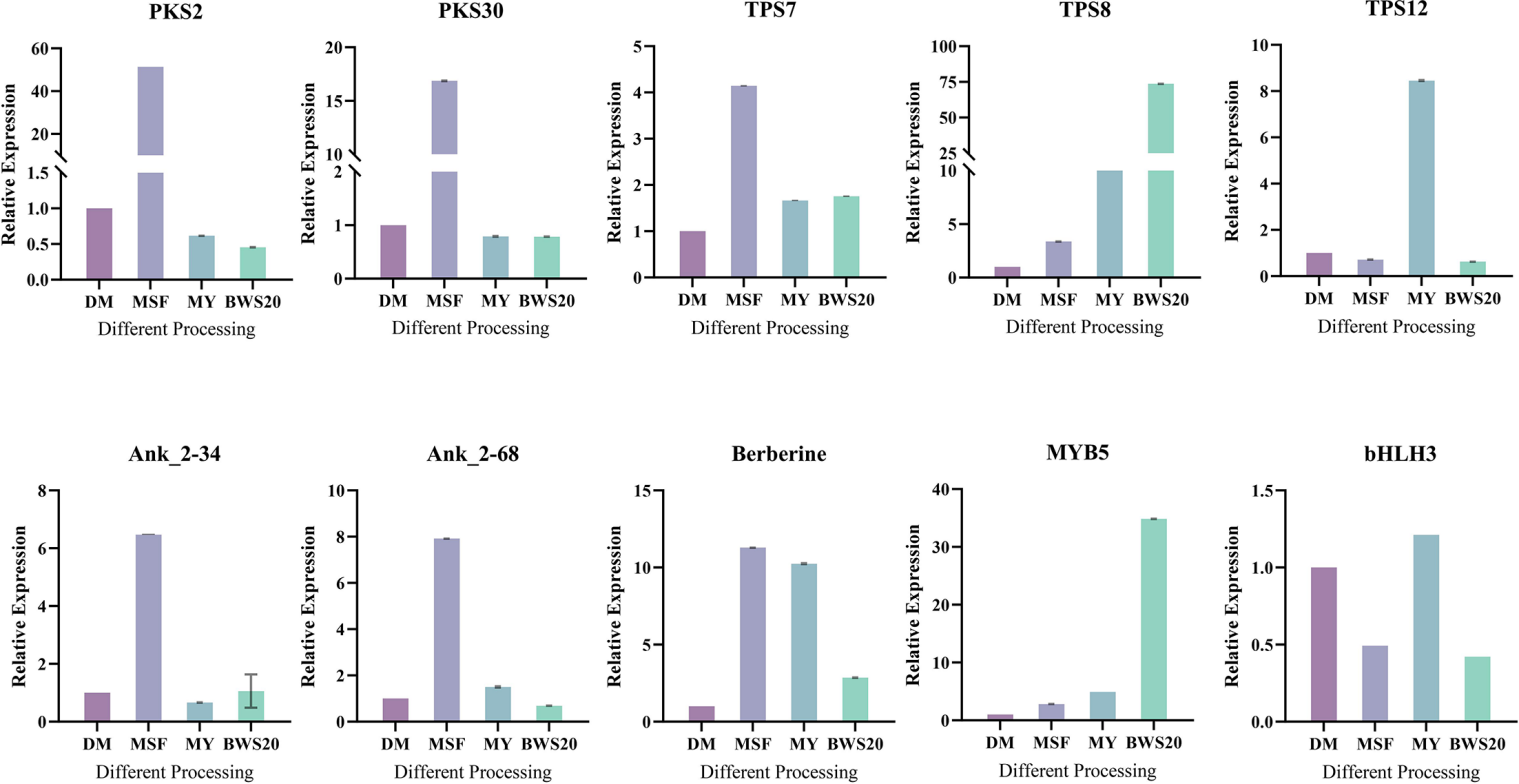
Relative expression of differentially expressed genes by quantitative real-time PCR. DM, mycelia cultured on rice medium; MSF, mycelia cultured on cassava flour medium; MY, mycelia cultured in yeast malt broth medium; BWS20, mycelia cultured in yeast malt broth medium supplemented with valproic acid.

Quantitative real-time PCR was utilized to analyse the expression of ten differential genes related to secondary metabolism in *Xylaria* sp. VDL4, validating the accuracy of the transcriptome sequencing results. The results indicated that the relative expression of these ten genes was consistent with the trends observed in the transcriptome data. Under solid cultivation conditions (MSF), the expression levels of *XyPKS2*, *XyPKS30*, *XyTPS7*, *XyBerberine*, *XyAnk2*-34 and *XyAnk2*-68 were up-regulated, while under liquid cultivation conditions (MY, BWS), *XyTPS8*, *XyTPS12*, *XybHLH3* and *XyMYB5* exhibited increased expression (Fig. 20). These findings suggest that cultivation conditions significantly influence the expression patterns of genes associated with SM synthesis in *Xylaria* sp. VDL4. Different cultivation conditions may promote the activation of specific metabolic pathways, thereby enhancing the synthesis of various metabolites.

### Discussion

*Xylaria* is capable of producing a diverse array of structurally novel and biologically active secondary metabolites, such as cytochalasin D [18], the monoterpenoid indole alkaloid 10-hydroxycamptothecin [96] and the polyketide 6-methylsalicylic acid. These compounds exhibit a wide range of biological activities, including anticancer, antioxidant, anti-inflammatory, immunomodulatory and antimicrobial effects. The rapid advancement of modern sequencing technologies has significantly propelled research into the genomes of *Xylaria* sp. VDL4 and other fungi. In this study, we integrated sequencing data from Oxford Nanopore Technologies and Illumina NovaSeq to successfully assemble the complete genome of *Xylaria* sp. VDL4. A comparative genomic analysis was conducted between *Xylaria* sp. VDL4 and 16 closely related species to examine differences in genomic features and secondary metabolite biosynthesis pathways. Additionally, evolutionary relationships and divergence times were investigated through gene family clustering and phylogenetic tree construction. The results revealed notable variations in genome size and GC content between *Xylaria* sp. VDL4 and its closely related species.

In the biosynthesis of fungal secondary metabolites, PKS and TPS play pivotal roles by catalysing diverse reactions to generate a wide array of secondary metabolites [97,98]. This study conducted genome mining on *Xylaria* sp. VDL4, identifying and elucidating putative secondary metabolite BGCs. Among these, a PR-PKS BGC associated with 6-methylsalicylic acid biosynthesis was identified, characterized by the domain arrangement KS-AT-DH-KR-ACP [99,100]. A phylogenetic tree was constructed for four PR-PKS genes sharing identical domain architectures with known 6-methylsalicylic acid-synthesizing PKS genes (Fig. S10). The results indicated that *XyPKS30* is related to 6-methylsalicylic acid biosynthesis. Additionally, three PKS genes with atypical domain arrangements – *XyPKS3* (KS-AT-MT-KR-ACP-TE), *XyPKS7* (KS-AT-DH-ACP-ACP-MT) and *XyPKS16* (KS-AT-DH-ACP-MT) – were identified in the genome of *Xylaria* sp. VDL4, which differs from the structural domains of known PKS genes; it was hypothesized that these three PKS genes might be novel PKS genes. In the *Xylaria* sp. VDL4 genomic data, a total of five *XyPKS-NRPS* were identified, and the construction of a phylogenetic tree and gene cluster comparison analysis showed that the multifunctional enzyme-encoding genes of *XyPKS-NRPS1* were consistent with Swnk, with a homology degree of 98%, and thus, it was hypothesized that *XyPKS-NRPS1* could catalyse the synthesis of swainsonine [90]. The BGC of *XyPKS-NRPS2* in this study contained four highly conserved genes essential for cytochalasin biosynthesis: PKS-NRPS, trans-ER, *α*/*β*-hydrolase and Diels–Alder cyclase [32,34, 44]. These genes are critical for constructing the core scaffold of cytochalasans, while other genes are modified after backbone synthesis [101,102]. *XyPKS-NRPS2* has high similarity with the reported BGC pyi [103] for the synthesis of pyrichalasin H in *Magnaporthe grisea* NI980, with 81% homology, and its structural domains are KS-AT-DH-MT-KR-ACP-C-A-TE. Since pyrichalasin H belongs to the cytochalasin family, we hypothesize that *XyPKS-NRPS2* may synthesize cytochalasin D or related compounds. In this study, seven other gene clusters known to be associated with cytochalasin synthesis were also comparatively analysed, and the results showed that although these gene clusters remained consistent in their structural domains, the modifier genes were significantly different. Horizontal shifts and partial deletions of modifier genes lead to differences in the final products [104]. *XyPKS-NRPS2* presumably synthesizes cytochalasin D using phenylalanine as a precursor, and other *Xylaria* species are capable of producing cytochalasans using other amino acids as precursors [105,107]. Under the culture conditions of this experiment, gene expression of *XyPKS-NRPS23* and *XyPKS-NRPS25* was low. Therefore, we hypothesize that these genes are silenced under the current culture conditions or that there are other regulatory mechanisms that have not yet been identified, and subsequent studies will test these hypotheses in depth.

Through phylogenetic analysis of the TPS genes from *Xylaria* sp. VDL4, we observed that *Xy-TPS4* clustered with squalene synthases, while *Xy-TPS11* likely utilizes GGPPS as a precursor to catalyse monoterpene biosynthesis. This suggests that the squalene synthase *Xy-TPS4* in *Xylaria* sp. VDL4 may participate in the biosynthesis of ganoderic acid Mf. Previous studies have elucidated key steps in ganoderic acid biosynthesis: for instance, CYP5150L8 was identified to catalyse the conversion of lanosterol to type I ganoderic acids, which are subsequently modified by various cytochrome P450 enzymes (CYPs) into type II ganoderic acids [104]. Notably, type II ganoderic acids often exhibit enhanced anti-tumour activity. Integrating current evidence with metabolic pathway analysis, we hypothesize that *Xy-TPS11* may employ GGPPS as a substrate to contribute to 10-hydroxycamptothecin biosynthesis. From a metabolic network perspective, the terpenoid backbones of both ganoderic acids and 10-hydroxycamptothecin derive from the MVA pathway, whereas their indole alkaloid moieties originate primarily from the shikimate pathway [40]. Of particular significance, 10-hydroxycamptothecin – a prominent monoterpenoid indole alkaloid [96] – has been extensively documented for its potent antitumour properties [22,25]. Recent years have witnessed significant advancements in camptothecin-related research: (1) studies on *Xylaria* sp. M71 revealed that salicylic acid treatment markedly up-regulates the expression of genes associated with 10-hydroxycamptothecin biosynthesis [108]. A potential camptothecin BGC was identified in *A. burnsii* NCIM 1409 [38]. TuanAnh *et al*. identified the key oxidase CYP81 through genome mining, which catalyses the regioselective hydroxylation of CPT to produce 11-hydroxycamptothecin [109]. In this study, we detected various camptothecin derivatives, including 10-hydroxycamptothecin, via untargeted metabolomics. Furthermore, multi-omics integration analysis of *Xylaria* sp. VDL4 enabled preliminary identification of candidate genes involved in camptothecin biosynthesis (Figs 16 and 17). These findings establish a crucial foundation for subsequent functional validation studies to elucidate the biosynthetic mechanisms of these pharmaceutically active compounds.

In recent years, a series of structurally novel compounds with multiple activities have been isolated and characterized from fungi through the OSMAC strategy [110]. Improving the growth environment by changing different media formulations promotes the growth of fungi, realizing the goal of ‘one fungus, many products’ (OSMAC) and triggering their metabolism outside of their native habitats to activate silent and critical BCGs [111,113]. It has been shown that, in addition to variations in nutrient content, different culture conditions can significantly affect metabolite production [114]. For instance, Luciana da S. *et al*. [115] cultivated *X. arbuscula* under varying culture conditions and conducted quantitative analysis of the produced cytochalasin D. The results demonstrated that cytochalasin D was most abundantly produced in wheat-based medium, followed by Czapek medium supplemented with 2% yeast extract. Multiple novel sesquiterpenes [26] were isolated from the co-culture fermentation of * X. hyperxylon* and *D. variisporum*. Yang *et al*. [116] significantly enhanced the production of aspochalasin D through fermentation condition optimization and metabolic engineering of the strain. Duan *et al*. isolated and characterized stereumamides A–D [117] from *Stereum hirsutum*, which exhibited antibacterial activity. Spirobrocazine A and brocazine G [118], displaying activity against *S. aureus*, were obtained from the marine endophytic fungus *Penicillium brocae* MA-231. Williams *et al*. treated *Diatrype* sp. with 5-azacytidine, yielding two new polyketides, lunalides A (7) and B (8). Yang *et al*. [119] employed metabolomics techniques to identify differential compounds in various medicinal parts of *Angelica sinensis*. The results revealed 18 distinct classes of metabolites with significant variations across different plant sections. In this study, metabolomic analysis clearly demonstrated differences in metabolite profiles among samples subjected to varying cultivation conditions. These differential metabolites help elucidate the association between the identified compounds and specific biological processes or physiological states. For instance, metabolites such as cytochalasin D and ganoderic acid Mf showed significantly increased concentrations in solid-state cultures supplemented with cassava flour. Conversely, 10-hydroxycamptothecin exhibited higher expression levels under rice-based cultivation conditions.

To further validate the findings of this study and advance subsequent research, future experiments will employ targeted LC-MS/MS analysis incorporating blank controls (non-inoculated medium) and authentic standards to eliminate medium interference and confirm the retention times and MS/MS fragmentation patterns of target compounds, thereby verifying the reliability of current identifications. Additionally, scale-up fermentation (e.g. 10–50 l) will be conducted based on the analytical methods established herein, coupled with bioassay-guided isolation to obtain sufficient quantities of target compounds for comprehensive spectroscopic characterization and in-depth bioactivity evaluation. This strategy not only validates existing discoveries through controlled experiments but also establishes a solid material foundation for subsequent investigations into metabolic pathway elucidation and structure-activity relationships.

Transcription factors are widely present in fungi and participate in the regulation of various biological processes [120]. For instance, Song *et al*. [121] identified a novel RNA-binding protein, CsdA, in filamentous fungi that regulates fungal secondary metabolism. They found that CsdA and the global regulator RsdA cooperatively modulate fungal secondary metabolism. In *Metarhizium acridum*, the C2H2-type transcription factor family is the most extensively reported group involved in pest virulence [122]. The bHLH transcription factor-encoding gene SRE1 plays distinct regulatory roles in the antagonism of *Rhodotorula kratochvilovae* strain IK726. Deletion of SRE1 enhanced the strain’s antagonistic ability against *Metarhizium* but reduced its antagonistic activity against *Myzus persicae* [123]. Meanwhile, HMG transcription factors may activate the expression of specific secondary metabolite gene clusters. Epigenetic modifications, such as histone acetylation and DNA methylation, play crucial roles in regulating fungal secondary metabolism, and HMG transcription factors may indirectly influence the synthesis of secondary metabolites by interacting with these modification mechanisms [124]. The binding motif of AflR (aflatoxin transcriptional activator) is present in the promoters of most sterigmatocystin and aflatoxin gene clusters. This transcription factor has been demonstrated to positively regulate both gene clusters [125,126], which aligns with the findings of this study. By integrating co-expression patterns under different culture conditions and predicting transcription factor binding sites, we hypothesize that the transcription factor *XyMYB2* directly modulates the transcription of *XyPKS18* through binding to its promoter region, thereby influencing the biosynthesis of secondary metabolites. The *XyPKS25* gene is regulated by the transcription factors *XyHMG1*, *XybHLH2* and *XyMYB3*. Furthermore, *XyMYB5* positively regulates the expression of *Xy-TPS7*, enhancing or activating the transcriptional processes of polyketide synthase and terpene synthase genes. This promotes enzyme synthesis and subsequently facilitates the production of polyketides and terpenoids.

### Conclusions

To elucidate the genomic characteristics of *Xylaria* sp. VDL4 and its secondary metabolite biosynthesis mechanisms. This study presents the first systematic investigation of this endophytic fungus using an integrated multi-omics strategy. The assembled genome sequence provides a foundational resource for future research. Metabolomic analysis revealed differential accumulation patterns of secondary metabolites under varying culture conditions, offering critical insights into their regulatory mechanisms. Transcriptomic profiling further explored the influence of different cultivation conditions on the expression of *XyPKS*, *XyTPS* and *XyTF*s genes, identifying specific conditions that may induce the expression of certain genes. Through co-expression network analysis and TF binding site prediction, we hypothesize that *XyTFs* may positively regulate the expression of *XyTPS* and *XyPKS* genes, thereby playing a pivotal role in the biosynthesis of secondary metabolites. By integrating multi-omics data, this study successfully identified key genes involved in secondary metabolite biosynthetic pathways. Specifically, we propose that *XyPKS-NRPS2* may contribute to the biosynthesis of cytochalasin D, while *Xy-TPS11* likely plays a crucial role in the biosynthetic pathway of 10-hydroxycamptothecin. Follow-up studies will employ molecular biology techniques, including gene knockout and heterologous expression, to functionally validate these candidate genes. In general, this study not only provides an important basis for an in-depth understanding of the synthesis mechanism of secondary metabolites of *Xylaria* sp. VDL4 but also lays a solid foundation for the validation of the function of the related genes and the application research.

## Supplementary material

10.1099/mgen.0.001658 Uncited Supplementary Material 1.

## Abbreviations

ACP: acyl carrier protein
AT: acyltransferase
BGCs: biosynthetic gene clusters
bHLH: basic helix–loop–helix
CPT: camptothecin
CYPs: cytochrome P450 enzymes
DH: dehydratase
FC: fold change
GA-Mf: ganoderic acid Mf
GGPPS: geranylgeranyl pyrophosphate synthetase
GO: Gene Ontology
HMG: high mobility group
HR-PKS: highly-reducing PKS
HSF: heat shock factor
KEGG: Kyoto Encyclopedia of Genes and Genomes
KNN: K-nearest neighbours
KR: ketoreductase
KS: ketosynthase
LC-MS/MS: liquid chromatography-MS/MS
MIAs: monoterpenoid indole alkaloids
6MSA: 6-methylsalicylic acid
MVA: mevalonate
MYB: myeloblastosis
NCBI: National Center for Biotechnology Information
NR: Non-Redundant
NR-PKS: non-reducing PKS
NRPS: non-ribosomal peptide synthase
OPLS-DA: orthogonal partial least squares-discriminant analysis
PCA: principal component analysis
P5CR: pyrroline-5-carboxylate reductase-like
PHI: Pathogen-Host Interactions database
PKS: polyketide synthase
PKS-NRPS: polyketide synthase-non-ribosomal peptide hybrid compounds
PR-PKS: partially reducing PKS
QC: quality control
SQS: squalene synthase
STR: strictosidine synthase
TE: thioesterase
TFIIB: Fungal Transcription Factor Domain
TFs: transcription factors
TPS: terpene synthase
TPS: terpene synthase
VIP: variable importance in projection
