# Supplementary material for: Genomic features and the transcriptional regulation of secondary metabolite biosynthesis of endophytic fungus Xylaria sp. VDL4 isolated from Vaccinium dunalianum
Source: Microb Genom. 2026 Apr 28;12(4):001658. doi: 10.1099/mgen.0.001658 (PMC13293291; doi:10.1099/mgen.0.001658)
Supplement: Uncited Supplementary Material 1. [file mgen-12-01658-s001.pdf]

Supporting Information

Table S1. Genomic Characteristics of the 16 Strains.
Table S2. Statistical table of clustering of *Xylaria* sp. VDL4, *Claviceps sorghi, Xylaria sp.* FL1042 and *Xylaria arbuscula* gene families.
Table S3. Complete polyketide synthase was obtained from *Xylaria* sp. VDL4.
Table S4. Complete terpene synthase was obtained from *Xylaria* sp. VDL4.
Table S5. Login IDs for genomes hypothesized to be associated with cytochalasin biosynthesis.
Table S6. Login IDs of genomes hypothesized to be related to 6MSA biosynthesis
Table S7. Primer list of genes for qRT-PCR.
Figure S1. Functional annotation of GO analysis of *Xylaria* sp. VDL4 gene encoded protein
Figure S2. Functional annotation of KEGG analysis of *Xylaria* sp. VDL4 gene encoded protein
Figure S3. Classification statistics of gene family clustering in the comparative genome of *Xylaria* sp. VDL4 with 16 other strains
Figure S4. *Xylaria* sp. VDL4 *Claviceps sorghi, Xylaria* sp. FL1042 and *Xylaria arbuscula* share the Venn map of the specific gene family.
Figure S5. Species phylogenetic tree of *Xylaria* sp. VDL4 with 16 other comparative genomes
Figure S6. QC sample correlation score plot
Figure S7. S-plot of the OPLS-DA
Figure S8. Phylogenetic Tree of PKS Proteins
Figure S9 Phylogenetic relationships of five PKS-NRPS in *Xylaria* sp. VDL4 with 15 strains of proximate PKS-NRPS biosynthesis core genes.
Figure S10. Phylogenetic relationships of four PR-PKSs in *Xylaria* sp. VDL4 with 13 strains of near-relative PR-PKS biosynthetic core genes.
Figure S11. Heat map of *XyPKS* and *XyTPS* gene expression interaction under different culture conditions
Figure S12. Heatmap of *XyTFs* gene expression interaction under different culture conditions

Table S1.

Strain	Accession Number	Total Length	Scaffold	GC Content (%)
Xylaria sp. FL1042	GCA_022495085.1	57 Mb	2489	39.5
Xylaria arbuscula	GCA_022385695.1	51.2 Mb	89	44.5
Xylaria bambusicola	GCA_022495145.1	45.7 Mb	233	45.5
Xylaria curta	GCA_022495235.1	43.9 Mb	269	47.5
Xylaria flabelliformis	GCA_022453505.1	41.6 Mb	198	48
Xylaria grammica	GCA_004353285.2	54.7 Mb	25	44
Xylaria sp. FL0064	GCA_022593225.1	42.8 Mb	79	47
Xylaria longipes	GCA_025201785.1	50.8 Mb	974	42.5
Xylaria cf. heliscus	GCA_022539335.1	44.9 Mb	72	47
Xylaria nigripes	GCA_022984845.1	34.6 Mb	64	48.5
Xylaria scruposa	GCA_022385635.1	46.6 Mb	589	45.5
Xylaria cubensis	GCA_022385715.1	44.8 Mb	333	46
Xylaria castorea	GCA_022495115.1	45.9 Mb	461	45.5
Xylaria venustula	GCA_022578775.1	50.8 Mb	392	43
Xylaria telfairii	GCA_022495065.1	46.9 Mb	1027	47
Xylaria hypoxylon	GCA_902806585.1	54.3 Mb	88	40.5

Table S2.

Species	Genes_number	Family_number	Unique_families
xylaria.sp	12,428	10,538	8
Claviceps sorghi	7,087	5,866	23
Xylaria sp. FL1042	12,015	10,375	6
Xylaria arbuscula	12,859	10,811	7

Table S3.

Protein name	ID	structural domain	classification
XyPKS1	Xsp00443.1	A-ACP-KS-AT-ACP-TE	PKS-NRPS
XyPKS2	Xsp01202.1	KS-AT-DH-MT-KR-ACP-C-A-TE	PKS-NRPS
XyPKS3	Xsp01328.1	KS-AT-MT-KR-ACP-TE	PR
XyPKS4	Xsp01486.1	KS-AT-DH-MT-ER-KR-ACP	HR
XyPKS5	Xsp01604.1	KS-AT-DH-MT-ER-KR-ACP	HR
XyPKS6	Xsp01994.1	KS-AT-DH-ER-KR-ACP	HR
XyPKS7	Xsp02434.2	KS-AT-MT-ACP	NR
XyPKS8	Xsp02974.1	KS-AT-DH-ER-KR-ACP	HR
XyPKS9	Xsp03127.1	KS-AT-KR-ACP	PR
XyPKS10	Xsp03372.1	KS-AT-DH-ER-KR-ACP	HR
XyPKS11	Xsp03713.1	KS-AT-DH-MT-ER-KR-ACP	HR

XyPKS12	Xsp03956.1	SAT-KS-AT-DH-ACP-ACP-TE	NR
XyPKS13	Xsp04260.1	KS-AT-DH-ER-KR-ACP	HR
XyPKS14	Xsp04645.1	KS-AT-KR-ACP	PR
XyPKS15	Xsp04918.1	KS-AT-DH-MT-KR-ACP-TE	PR
XyPKS16	Xsp04972.1	KS-AT-DH-ACP-MT	NR
XyPKS17	Xsp05372.2	SAT-KS-AT-DH-ACP-ACP-TE	NR
XyPKS18	Xsp06248.1	KS-AT-DH-KR-ACP	PR
XyPKS19	Xsp06288.1	KS-AT-DH-ER-KR-ACP	HR
XyPKS20	Xsp06322.1	SAT-KS-AT-DH-ACP-MT-TE	NR
XyPKS21	Xsp06627.1	KS-AT-DH-MT-ER-KR-ACP	HR
XyPKS22	Xsp06784.1	KS-AT-DH-ACP-ACP-MT	NR
XyPKS23	Xsp07321.1	KS-AT-DH-MT-KR-ACP-C-A-TE	PKS-NRPS
XyPKS24	Xsp07808.1	SAT-KS-AT-PT-ACP-MT	NR
XyPKS25	Xsp08052.1	KS-AT-DH-MT-KR-ACP-C-A-TE	PKS-NRPS
XyPKS26	Xsp08190.1	SAT-KS-AT-DH-ACP-TE	NR
XyPKS27	Xsp08290.1	SAT-KS-AT-DH-ACP-TE	NR
XyPKS28	Xsp08633.1	KS-AT-DH-ER-KR-ACP	HR
XyPKS29	Xsp08768.1	KS-AT-DH-KR-ACP	PR
XyPKS30	Xsp08977.1	KS-AT-DH-KR-ACP	PR
XyPKS31	scaffold9.t231	KS-AT-DH-ER-KR-ACP-C-A	PKS-NRPS

Table S4.

Protein name	ID	classification
Xy-TPS1	Xsp01042.1	Terpene synthases
Xy-TPS2	Xsp01195.1	Terpene synthases
Xy-TPS3	Xsp01595.1	prenyltransferases
Xy-TPS4	Xsp01668.1	Squalene synthase
Xy-TPS5	Xsp02223.1	Squalene cyclase
Xy-TPS6	Xsp02499.1	Squalene cyclase
Xy-TPS7	Xsp04341.1	Terpene cyclase
Xy-TPS8	Xsp05942.1	Terpene synthases
Xy-TPS9	Xsp06434.1	Terpene synthases
Xy-TPS10	Xsp06452.1	Terpene synthases
Xy-TPS11	Xsp07241.1	Monoterpene synthases
Xy-TPS12	Xsp07902.1	Terpene synthases
Xy-TPS13	Xsp07978.1	Terpene synthases
Xy-TPS14	Xsp08447.1	polyprenyl synthetase
Xy-TPS15	Xsp08644.1	Terpene cyclase
Xy-TPS16	Xsp09508.1	prenyltransferases
Xy-TPS17	Xsp09933.1	Terpene cyclase

Table S5.

Strain	Accession Number
Parastogonospora nodorum SN15	GCA_016801405.1
Penicilliumt expansun	GCF_000769745.1
Aspergillus.flavipes CNL-338	MT586757.1
Aspergillus clavatus NRRL	GCA_000002715.1
Chaetomium globosum CBS148.51	GCA_000143365.1
Magnaporthe grisea NI980	GCA_004355905.1
Pyricularia oryzae Guy 11	GCA_002368485.1

Table S6.

Strain	Accession Number
Penicillium griseofulvum	GCA_001561935.1
Aspergillus terreus NlH2624	GCA_000149615.1
Macrophomina phaseolina	GCA_000302655.1
Nemania sp. FL0031	GCA_022432375.1
Xylaria telfairii	GCA_022495065.1

Table S7.

Primer Name	Sequence（5'-3'）
Tubulin alpha-1A-F	CTATACAGTTGGCAAGGAGC
Tubulin alpha-1A-R	GAGTTGTATGGCTCGACGAC
XyPKS2-F	CTATGCACCAAGCTGTGCAA
XyPKS2-R	ATCAGGCACTCAATGTGGTC
XyPKS30-F	ATCACTATCGGGCTCGGATA
XyPKS30-R	AGATCCCTCGGGTGAAGTAG
XyAnk_2-34-F	CGGCCACATAGAGATCATGT
XyAnk_2-34-R	ATGGCTATCTGCTGTGCAGT
XyTPS7-F	CTGACGATATCATGGACAGC
XyTPS7-R	AGATCGCACAGTTGTCCGAG
XyAnk_2-68-F	TCGACGATGTCGACCTTGAC
XyAnk_2-68-R	TCGGAAGCTCTGCGCCAGTT
XyBerberine-F	AGCAGTCGCGATCAAGACTG
XyBerberine-R	TACATGACCTCCGAGATGTA
XyTPS8-F	AACCAAGGAGCCTTGAACGC
XyTPS8-R	GCGTGGTCGACATCTTCCAT
XyTPS12-F	GTGACTCCTCCAACGATGAG
XyTPS12-R	AGTGAACGTGGTGTTGTCGG
XyMYB5-F	GAGTGACAACGCCGTCAAGA
XyMYB5-R	GCGATCGGCCTGGAATACGT
XybHLH3-F	GACTACAGCTCGCAGGACAC
XybHLH3-R	CGCTTGTCACTGGCGGTAGA

Figure S1.

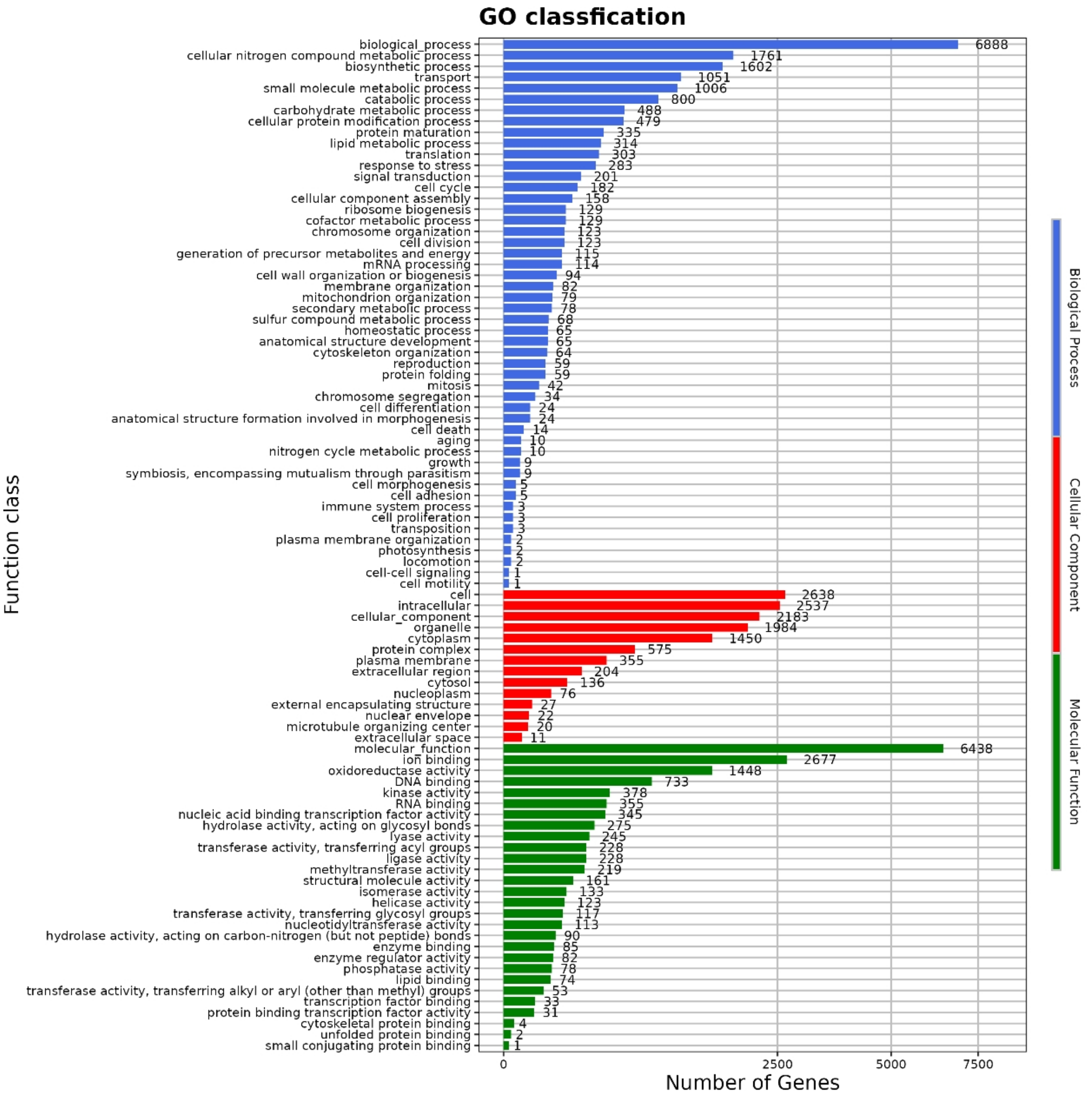
GO classfication
Function class
Number of Genes
0
2500
5000
7500
Biological Process
Cellular Component
Molecular Function
biological_process 6888
cellular nitrogen compound metabolic process 1761
biosynthetic process 1602
transport 1051
small molecule metabolic process 1006
catabolic process 800
carbohydrate metabolic process 488
cellular protein modification process 479
protein maturation 335
lipid metabolic process 314
translation 303
response to stress 283
signal transduction 201
cell cycle 182
cellular component assembly 158
ribosome biogenesis 129
cofactor metabolic process 129
chromosome organization 123
cell division 123
generation of precursor metabolites and energy 115
mRNA processing 114
cell wall organization or biogenesis 94
membrane organization 82
mitochondrion organization 79
secondary metabolic process 78
sulfur compound metabolic process 68
homeostatic process 65
anatomical structure development 65
cytoskeleton organization 64
reproduction 59
protein folding 59
mitosis 42
chromosome segregation 34
cell differentiation 24
anatomical structure formation involved in morphogenesis 24
cell death 14
aging 10
nitrogen cycle metabolic process 10
growth 9
symbiosis, encompassing mutualism through parasitism 9
cell morphogenesis 5
cell adhesion 5
immune system process 3
cell proliferation 3
transposition 3
plasma membrane organization 2
photosynthesis 2
locomotion 2
cell-cell signaling 1
cell motility 1
cell 2638
intracellular 2537
cellular_component 2183
organelle 1984
cytoplasm 1450
protein complex 575
plasma membrane 355
extracellular region 204
cytosol 136
nucleoplasm 76
external encapsulating structure 27
nuclear envelope 22
microtubule organizing center 20
extracellular space 11
molecular_function 6438
ion binding 2677
oxidoreductase activity 1448
DNA binding 733
kinase activity 378
RNA binding 355
nucleic acid binding transcription factor activity 345
hydrolase activity, acting on glycosyl bonds 275
lyase activity 245
transferase activity, transferring acyl groups 228
ligase activity 228
methyltransferase activity 219
structural molecule activity 161
isomerase activity 133
helicase activity 123
transferase activity, transferring glycosyl groups 117
nucleotidyltransferase activity 113
hydrolase activity, acting on carbon-nitrogen (but not peptide) bonds 90
enzyme binding 85
enzyme regulator activity 82
phosphatase activity 78
lipid binding 74
transferase activity, transferring alkyl or aryl (other than methyl) groups 53
transcription factor binding 33
protein binding transcription factor activity 31
cytoskeletal protein binding 4
unfolded protein binding 2
small conjugating protein binding 1

Figure S2.

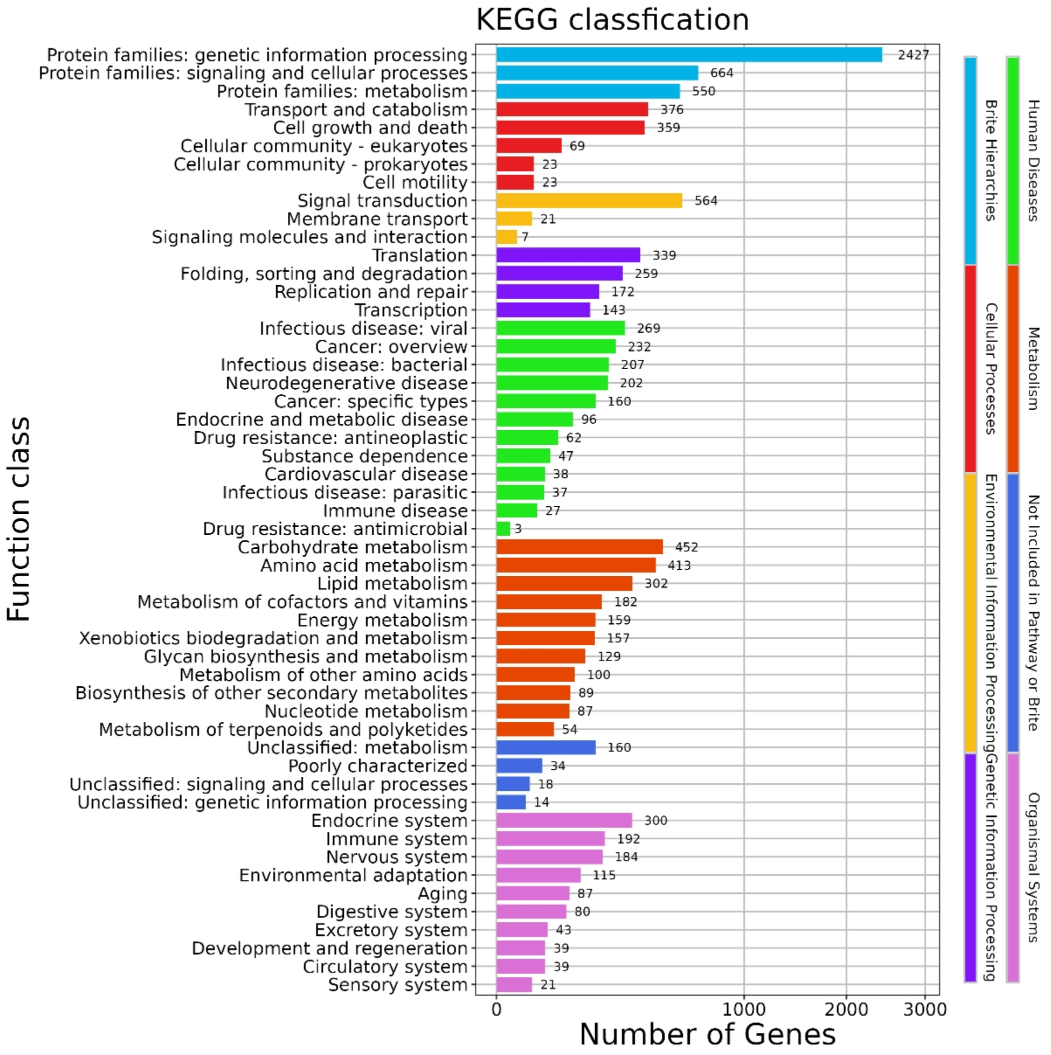

Figure S3.

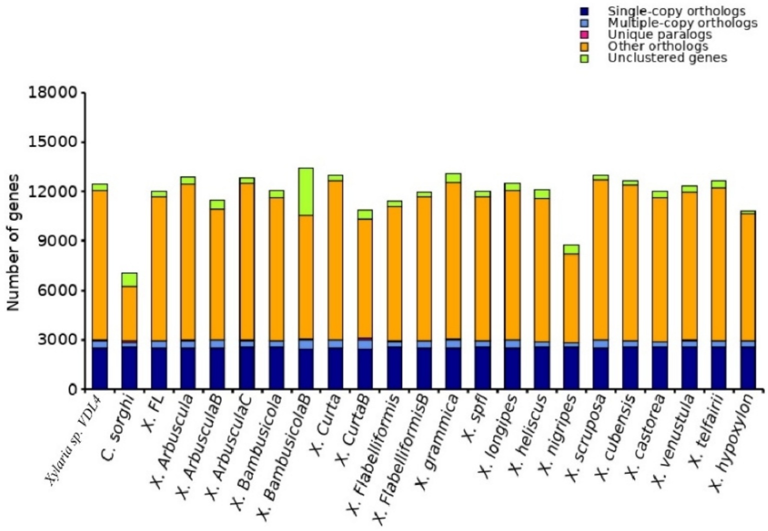

Figure S4.

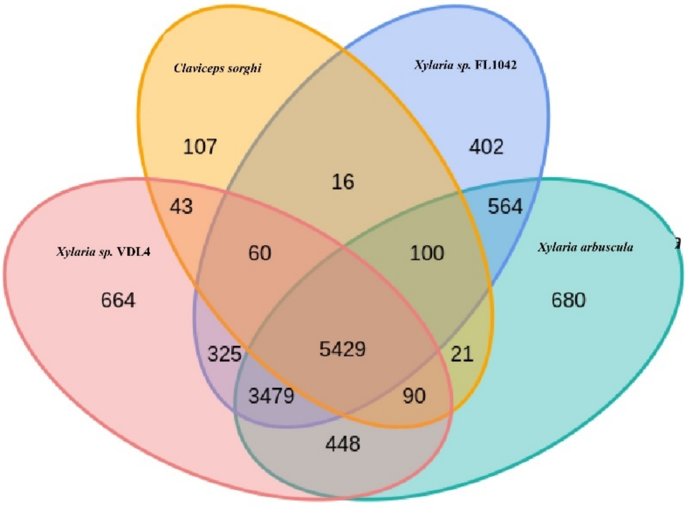

Figure S5.

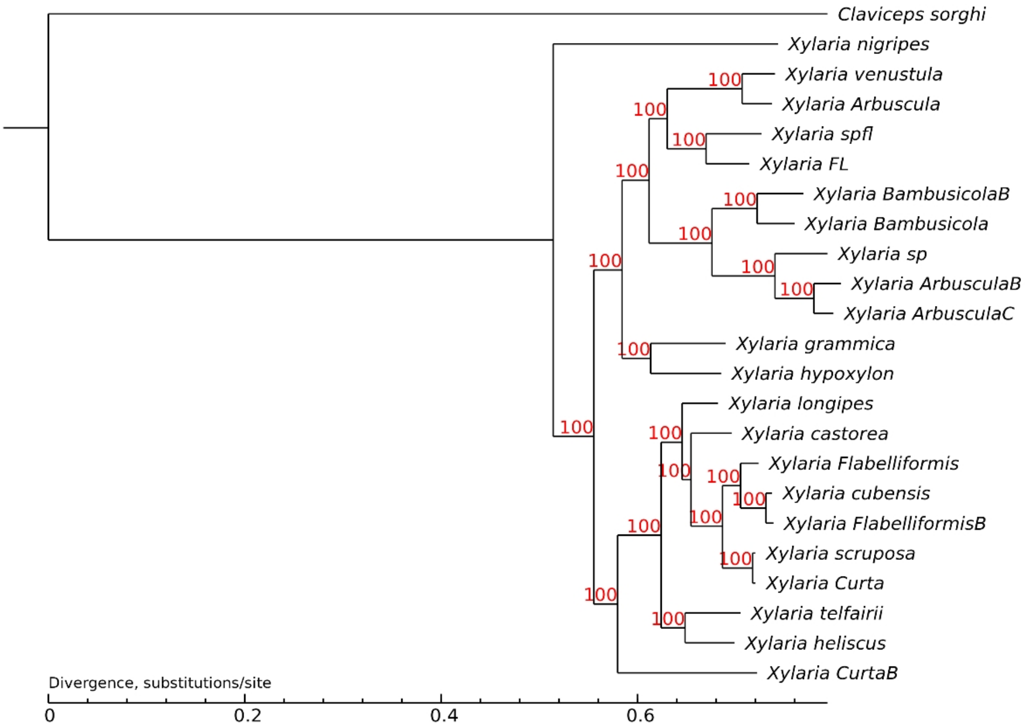

Figure S6.

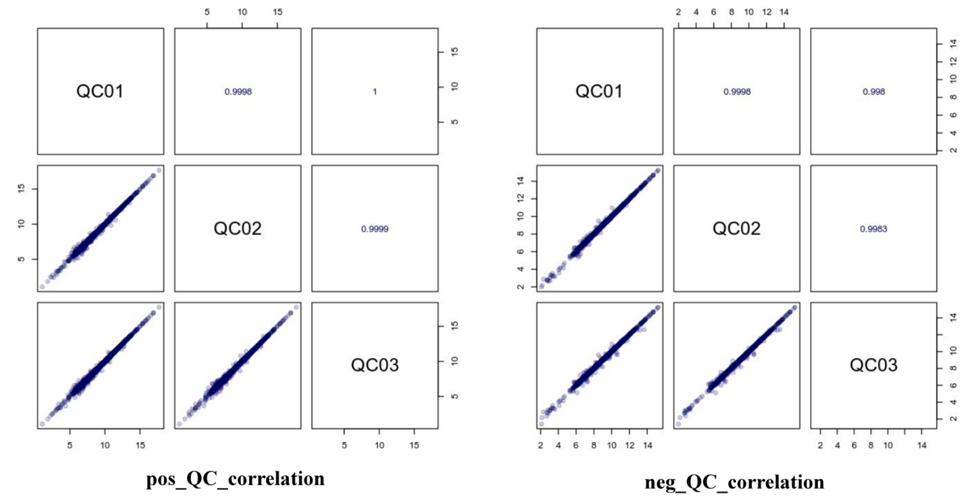

Figure S7.

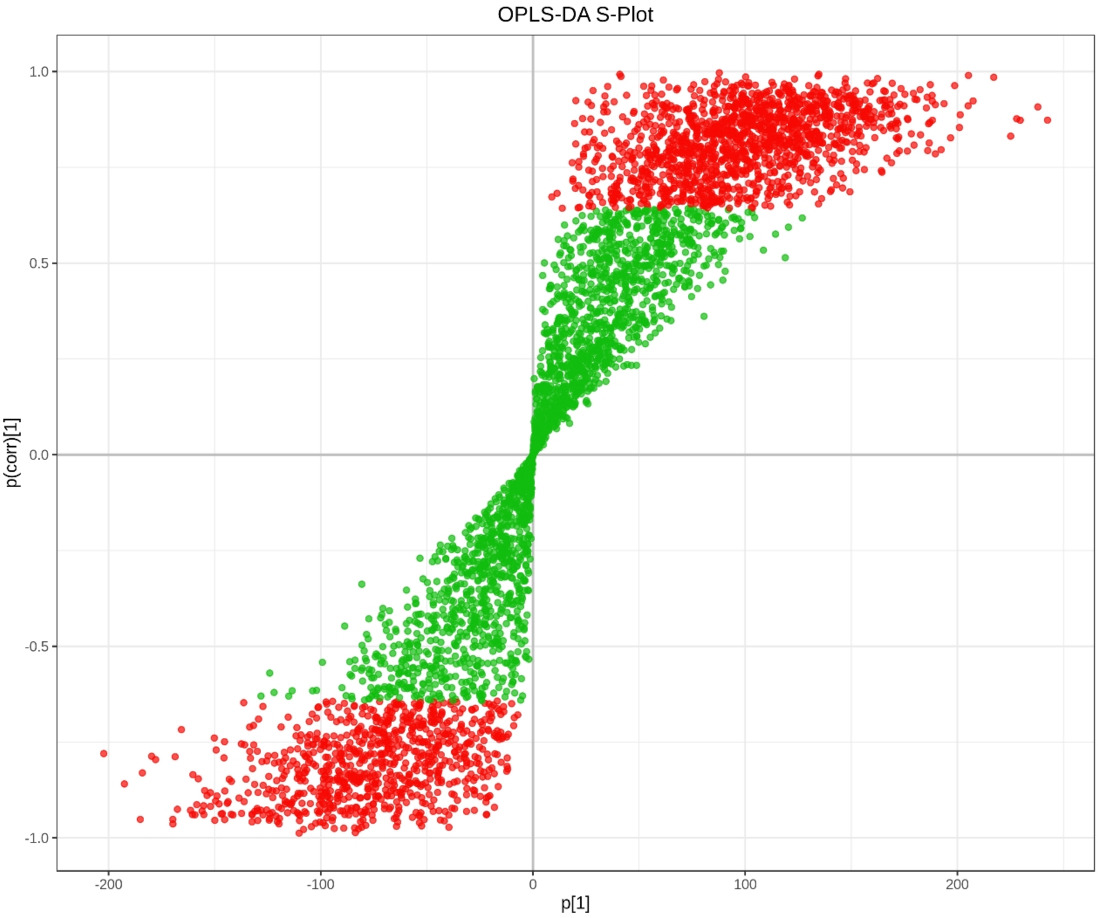

Figure S8.

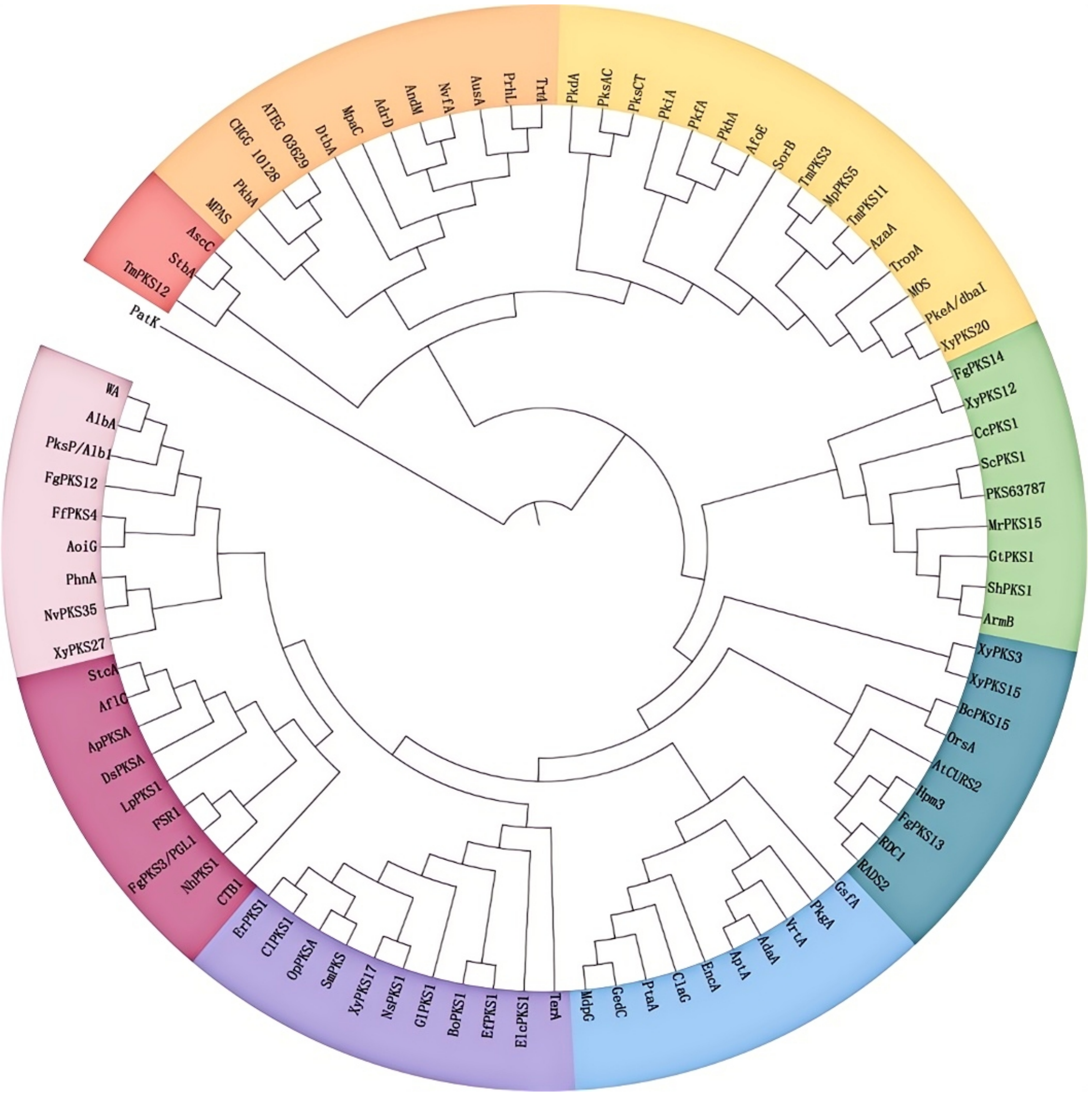

Figure S9

Figure S10.

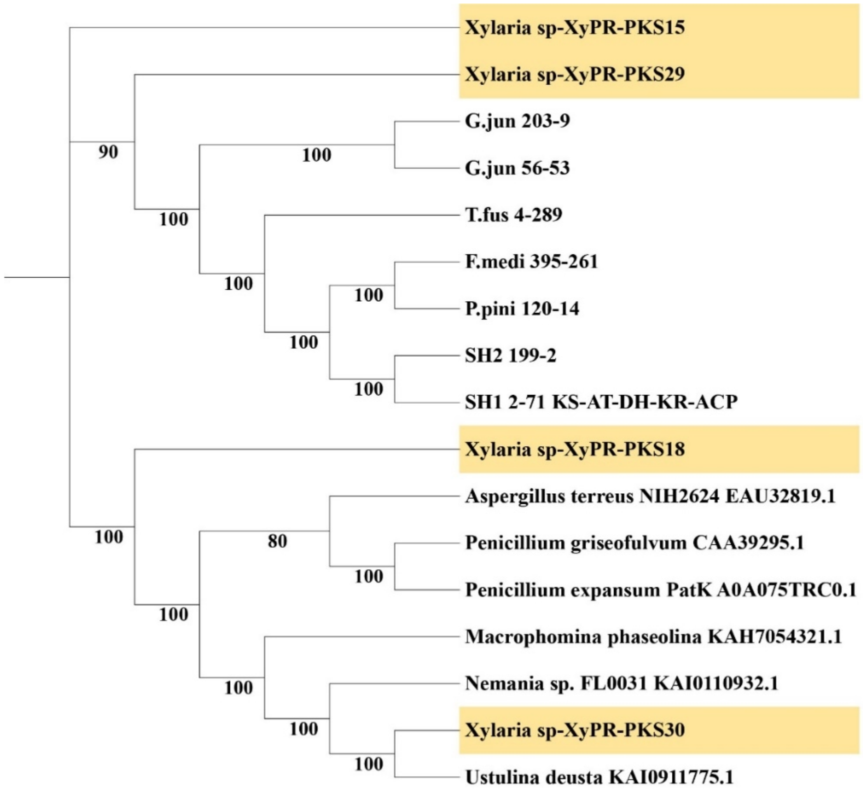

Xylaria sp-XyPR-PKS15
Xylaria sp-XyPR-PKS29
G.jun 203-9
G.jun 56-53
T.fus 4-289
F.medi 395-261
P.pini 120-14
SH2 199-2
SH1 2-71 KS-AT-DH-KR-ACP
Xylaria sp-XyPR-PKS18
Aspergillus terreus NIH2624 EAU32819.1
Penicillium griseofulvum CAA39295.1
Penicillium expansum PatK A0A075TRC0.1
Macrophomina phaseolina KAH7054321.1
Nemania sp. FL0031 KAI0110932.1
Xylaria sp-XyPR-PKS30
Ustulina deusta KAI0911775.1
90
100
100
100
100
100
100
100
80
100
100
100
100
100

Figure S11.

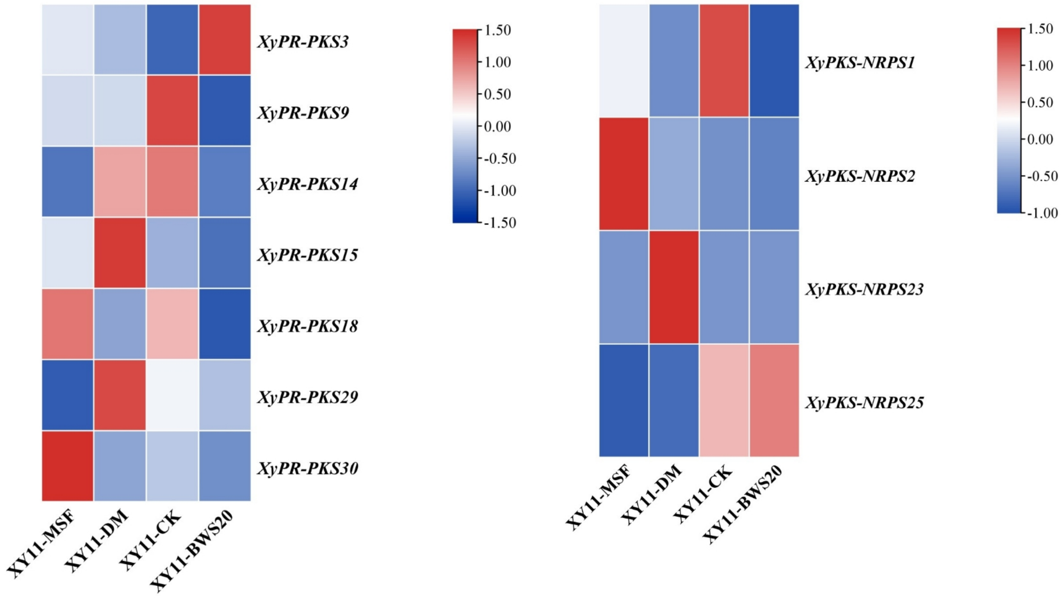

XyPR-PKS3
XyPR-PKS9
XyPR-PKS14
XyPR-PKS15
XyPR-PKS18
XyPR-PKS29
XyPR-PKS30
XY11-MSF
XY11-DM
XY11-CK
XY11-BWS20
1.50
1.00
0.50
0.00
-0.50
-1.00
-1.50
XyPKS-NRPS1
XyPKS-NRPS2
XyPKS-NRPS23
XyPKS-NRPS25
XY11-MSF
XY11-DM
XY11-CK
XY11-BWS20
1.50
1.00
0.50
0.00
-0.50
-1.00

a

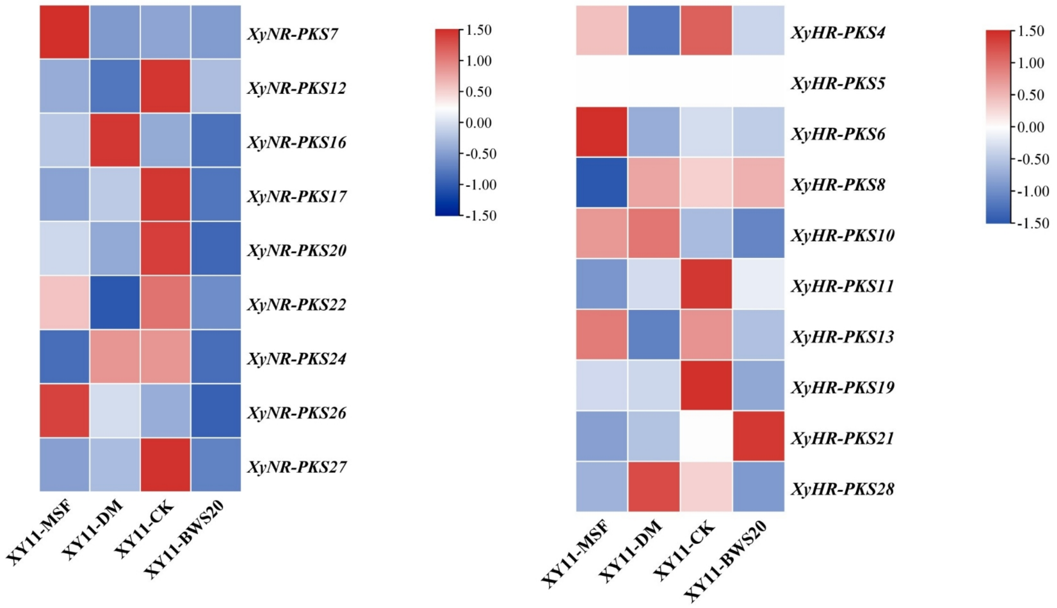
XyNR-PKS7
XyNR-PKS12
XyNR-PKS16
XyNR-PKS17
XyNR-PKS20
XyNR-PKS22
XyNR-PKS24
XyNR-PKS26
XyNR-PKS27
XY11-MSF
XY11-DM
XY11-CK
XY11-BWS20
1.50
1.00
0.50
0.00
-0.50
-1.00
-1.50
XyHR-PKS4
XyHR-PKS5
XyHR-PKS6
XyHR-PKS8
XyHR-PKS10
XyHR-PKS11
XyHR-PKS13
XyHR-PKS19
XyHR-PKS21
XyHR-PKS28
XY11-MSF
XY11-DM
XY11-CK
XY11-BWS20
1.50
1.00
0.50
0.00
-0.50
-1.00
-1.50

b

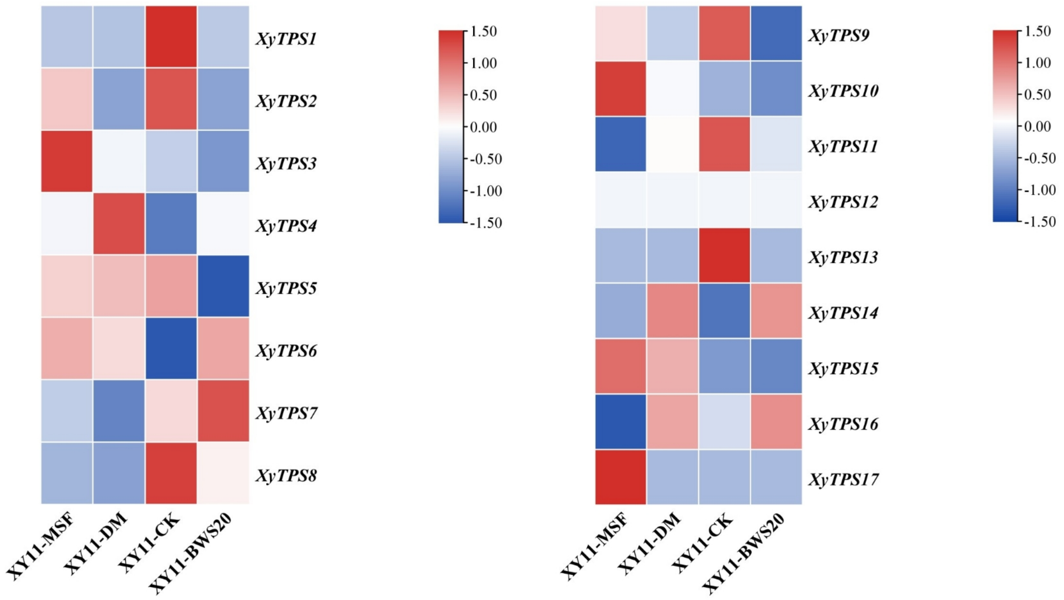
XyTPS1
XyTPS2
XyTPS3
XyTPS4
XyTPS5
XyTPS6
XyTPS7
XyTPS8
XY11-MSF
XY11-DM
XY11-CK
XY11-BWS20
1.50
1.00
0.50
0.00
-0.50
-1.00
-1.50
XyTPS9
XyTPS10
XyTPS11
XyTPS12
XyTPS13
XyTPS14
XyTPS15
XyTPS16
XyTPS17
XY11-MSF
XY11-DM
XY11-CK
XY11-BWS20
1.50
1.00
0.50
0.00
-0.50
-1.00
-1.50

c

Figure S12.

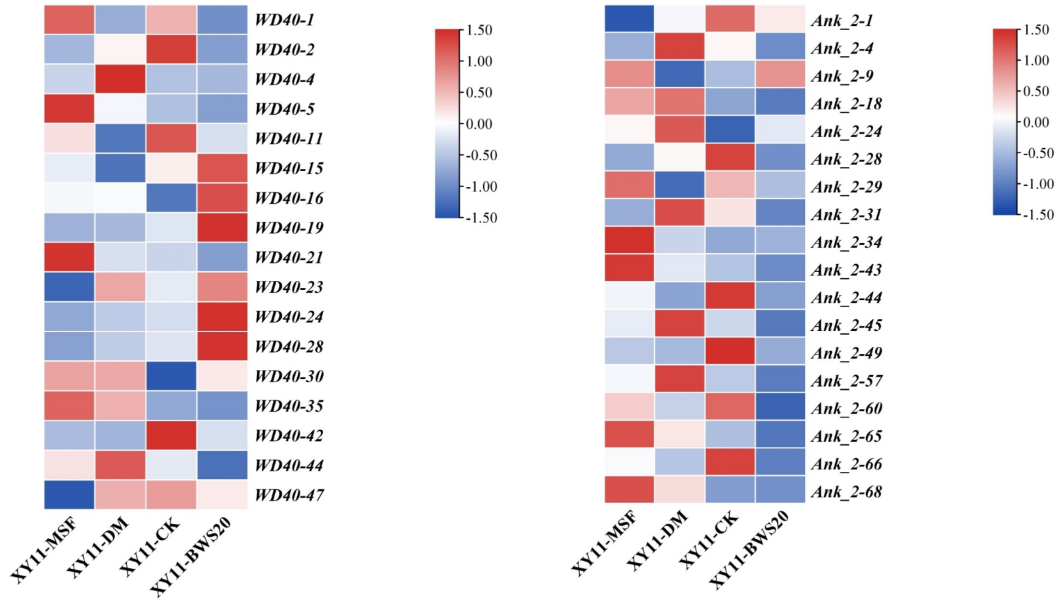

a

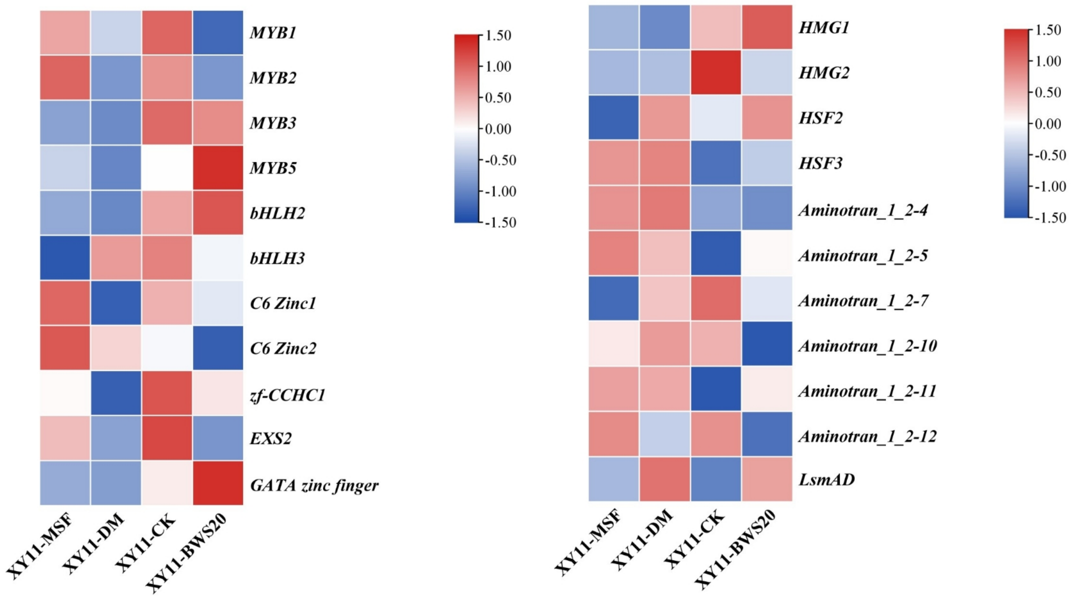

b
